# Beyond Co‐Occurrence: Multi‐Scale Evidence for Segregation‐Dominated Plant Networks in the French Alps

**DOI:** 10.1111/ele.70393

**Published:** 2026-05-01

**Authors:** Matthias Rohr, Alexandre Wendling, Tamara Münkemüller, Dominique Gravel, Clovis Galiez, Julien Renaud, Orchamp Consortium, Wilfried Thuiller

**Affiliations:** ^1^ Univ. Grenoble Alpes, Univ. Savoie Mont Blanc, CNRS, LECA Grenoble France; ^2^ Univ. Grenoble Alpes, Grenoble INP, CNRS, LJK Grenoble France; ^3^ Département de Biologie Université de Sherbrooke Sherbrooke Quebec Canada

**Keywords:** community ecology, environmental gradient, functional traits, mountain ecosystems, spatial patterns

## Abstract

Understanding how plants influence each other's spatial distribution is pivotal not only for interpreting current communities, but also for anticipating their responses to global changes. The combination of high‐resolution, multi‐scale sampling and novel statistical frameworks now enables us to identify species aggregations and segregations within their local co‐occurrences. By applying this approach to approximately 800 plant species and their communities across the French Alps, we discovered that local species associations are dependent on soil acidity and nitrogen rather than climate. By building a regional network from these associations, we identified a centralised core comprising a few dominant, stress‐tolerant graminoids and shrubs with high leaf dry matter content and no unique functional roles. Our findings demonstrate that plant community assembly is less dependent on random co‐occurrence and more dependent on segregation around a few dominant, stress‐tolerant species, with soil conditions modulating the outcome of local associations.

## Graphical abstract

Using high‐resolution spatial data and a multi‐scale statistical framework, we disentangle plant co‐occurrence from true spatial associations across alpine communities in the French Alps. We discovered that local species associations are dependent on soil acidity and nitrogen rather than temperature. By building a global network from these associations, we identified a centralised core comprising a few dominant, stress‐tolerant graminoids and shrubs. These findings highlight segregation around key structural species as a major driver of alpine community assembly, with soil conditions modulating local interaction outcomes.

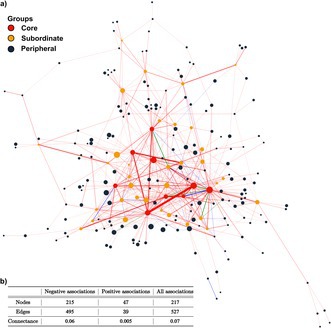

## 1 Introduction

Understanding how biotic interactions shape the structure of ecological communities remains a cornerstone of community ecology (Thompson et al. 2012; Thuiller et al. 2013). In diverse plant communities, direct observation or experimental manipulation of these interactions is often impracticable. Perennial life histories and high local richness make manipulative experiments logistically prohibitive, as community complexity scales quadratically with species number (Chalmandrier et al. 2022). Consequently, ecologists frequently use proxy approaches to infer pairwise interactions from field data (Blanchet et al. 2020; Morales‐Castilla et al. 2015), with analyses of spatial associations becoming particularly popular (Chillo et al. 2021; Losapio et al. 2018; Saiz et al. 2018). Specifically, spatial aggregation between species pairs (individuals spatially closer than expected by chance) is generally interpreted as evidence for facilitation, whereas spatial segregation is interpreted as evidence for competition (Freilich et al. 2018). Despite the valuable insights gained from pairwise spatial analyses (Losapio et al. 2021; Saiz et al. 2018), two critical knowledge gaps remain. First, we lack understanding of how spatial associations, and thus inferred interactions, vary across broad environmental gradients (e.g., temperature or disturbance, Bektaş et al. 2023; Pellissier et al. 2018). Second, it remains unclear whether all species contribute equally to pairwise spatial aggregations and segregations, or whether dominant species disproportionally influence other species, thereby contributing more to the overall spatial associations within communities.

Pairwise spatial aggregations and segregations can be integrated into networks and then jointly analysed (Losapio et al. 2019). By representing species as nodes and their positive or negative associations as signed links, network theory can quantify emergent properties (e.g., degree, betweenness and centralization) and link them to community functioning (Delmas et al. 2019). In species‐rich plant assemblages, we anticipate strong heterogeneity of species' network positions: a few highly connected ‘hub’ species, often dominant, should occupy central roles, while many species form sparsely connected peripheries (González et al. 2010). Hub species, whose abundance are likely influenced by environmental drivers, and modulate the establishment of peripheral species (Arnillas and Cadotte 2019), thus generating predictable network structure, for example a high degree centrality (Chillo et al. 2021; Delmas et al. 2019). Identifying such keystone or foundation species and their effect on peripheral species is essential for diagnosing community‐wide responses to environmental change.

To date, studies on spatial association networks of diverse plant communities are rare, often focusing on single sites (Losapio et al. 2018) or overlooking how associations vary across different environmental conditions (Losapio et al. 2021; Saiz et al. 2018). Yet, these conditions are decisive for community assembly and network topology. For example, the *stress–gradient hypothesis* (Callaway et al. 2002) predicts a shift from competition to facilitation along an abiotic gradient (Callaway et al. 2002). If valid, this should manifest in network topology as more positive associations and weaker segregation under stressful conditions (Chillo et al. 2021; Pellissier et al. 2018).

Both a species' response to the abiotic context and its effect on neighbours are mediated by its *functional traits*—morphological, physiological or phenological attributes that influence performance (Violle et al. 2007). Leaf traits, for instance, reliably place plants along Grime's CSR (Competition–Stress tolerance–Ruderal) strategy spectrum (Grime 1974; Pierce et al. 2013). Integrating trait information with network metrics thus provides a route to identify which traits characterise dominant, highly connected species and test whether these species are functionally distinct from their local neighbourhoods.

Here, we combine an extensive, high‐resolution data set of spatially explicit plant communities across the French Alps with a recently developed statistical framework that partitions species associations into three nested components: (i) co‐occurrence at plot level (i.e., shared presences along a 30 m reading line, chosen to maximise homogeneity of vegetation and abiotic conditions), (ii) local spatial association at plot level (plot level evidence of spatial association) and (iii) regional spatial association, combining local evidence across the whole French Alps. This decomposition allows us to construct both an average regional network and hundreds of local association networks, thereby retaining local idiosyncrasies while quantifying and generalising overarching patterns. We address the following three questions:

- What is the structure of the regional spatial association network? In our species rich alpine grasslands, we expect intense competitive interactions, which should result in a preponderance of negative (segregating) spatial associations and a centralised topology dominated by a few highly connected species.
- How do local associations change along abiotic gradients? Following the stress–gradient hypothesis, we predict that benign, highly productive sites will exhibit stronger segregation driven by few dominant species in contrast to stressful sites, where facilitation and aggregation should be more prevalent.
- Which species (or species groups) act as core species, and what are their functional attributes? To characterise these species and how they differ from other species, we assess whether core species share convergent CSR strategies, and whether they are functionally distinct or common relative to their local communities.

By integrating network theory, trait ecology and multi‐scale community data, our study provides a comprehensive, scale‐explicit framework to unravel how environmental context and functional strategies jointly shape plant–plant association networks in species‐rich ecosystems.

## 2 Material and Methods

We combined (i) a uniquely fine‐scale, spatially explicit vegetation data set collected across steep elevational and environmental gradients in the French Alps; (ii) a recently developed statistical procedure that partitions spatial association signals across nested spatial scales (regional vs. local); and (iii) network and trait‐based analyses. Below we describe each component in turn.

### 2.1 Data

#### 2.1.1 Plant Sampling Design

The vegetation data stem from the Orchamp long‐term biodiversity observatory (Thuiller et al. 2024), which monitors biodiversity along multiple elevational gradients in the French mountains (Alps, Pyrénées and Corsica). We considered 30 gradients spanning on average 850 m in elevation (altitude range: 280–2940 m) located in the French Alps and surveyed between 2016 and 2024. All gradients cover different elevational belts and most of them all the typical habitats found along elevational gradients including mixed and coniferous forests, shrublands and subalpine and alpine grasslands (Calderón‐Sanou et al. 2024; Thuiller et al. 2024).

Each gradient comprises four to nine permanent 900 m^2^ plots, spaced by *∼* 200 m of elevation. Plot positions were chosen to maximise the homogeneity of local vegetation and abiotic factors, including orientation and edaphic conditions. Several gradients were sampled multiple times. To avoid non‐independent sampling, only the first record per gradient was retained. As a result, we considered data from 148 plots and analysed their spatial structure. We analysed in total 796 vascular plant species, only excluding a few juvenile phanerophytes (4947 individuals, *∼* 5% of the data), as we did not have access to their juvenile trait information.

In every plot, vegetation was sampled with the high‐resolution point–intercept method: two parallel 30 m lines (50 cm apart) were sampled at 20 cm intervals, producing 300 pinpoints per plot. Every individual with a pinpoint contact was recorded and identified. Importantly, in the botanical protocol, adult trees are not surveyed. This design distinguishes co‐occurrence (species present in the same 30 × 0.5 m plot, thus sharing similar environmental preferences) from co‐location (individual plant contacting the same pinpoint, coexisting at very fine spatial scales), a prerequisite for the multi‐scale association analysis introduced below.

#### 2.1.2 Climatic and Edaphic Data

We extracted 30‐year means (1992–2022) of six bioclimatic variables from the SAFRAN–SURFEX/ISBACrocus reanalysis (Durand et al. 2009; Vernay et al. 2021), which account for weather and snow conditions based on large‐scale topography. For each plot, we calculated the following variables: mean annual precipitation, mean daily temperature and growing degree days (GDD) which are related to growing‐season length (Choler 2018); minimum temperature of the coldest month and freezing degree days (FDD) capturing freezing stress; and total annual evapotranspiration (ETP), indicative of water–energy dynamics (Kreft and Jetz 2007; Li et al. 2022). GDD and FDD were computed as the cumulative sum of average daily temperatures above and below 0°C, respectively. Temperature data were taken at 5 cm below ground to reflect the thermal conditions experienced by perennial plant roots (Choler 2018).

Soils were sampled in three 2 *×* 2 m subplots distributed along the vegetation survey lines. In each subplot, 1 kg of soil was collected from multiple cores at a depth of *∼* 15 cm (excluding litter). We measured total nitrogen (%N), total carbon (%C), the C:N ratio, soil organic matter and pH (details in Thuiller et al. 2024). The three soil measurements were averaged to obtain a single value for each plot. Climatic and edaphic data were available for 141 plots in 26 gradients; subsequent environment‐dependent analyses were therefore restricted to this subset.

#### 2.1.3 Trait Data

We compiled five traits representing both resource economics and competitive ability: specific leaf area (SLA), leaf dry matter content (LDMC), leaf area (LA), vegetative height and seed mass (Bektaş et al. 2023; Kunstler et al. 2012; Pierce et al. 2017; Wright et al. 2004). Primary data were taken from the Androsace database (https://originalps.osug.fr/) and completed with the TRY database (Kattge et al. 2020). We had complete measured data for 422 species; remaining gaps (11%–34% depending on trait, LA: 34%, SLA: 24%, vegetative height: 11%, seed mass: 25%, LDMC: 15%) were imputed with Rphylopars (Goolsby et al. 2017). A maximum likelihood frequentist method that uses a phylogeny and a sparse trait matrix to estimate simultaneously the across‐species (phylogenetic) and within‐species trait covariance (Goolsby et al. 2017; Johnson et al. 2021), using the alpine Flora phylogeny (PhyloAlps project, Lavergne et al. 2025). The final trait matrix covered 696 species (86% of the flora); species with no trait values (usually species with very low frequencies across the area) were retained for network construction but excluded from trait‐based analyses. To assess potential bias from trait imputation, we repeated functional analyses using only the 422 species with complete trait information. Results were unchanged, indicating that trait imputation did not bias the analyses (Appendix S4.1).

### 2.2 Inferring Spatial Association Network

#### 2.2.1 Local Tests of Spatial Associations

We tested whether the observed number of co‐location (i.e., shared pinpoints, $n_{\mathrm{ijs}}$) differed from a random allocation of individuals to the *N*_*s*_ = 300 pinpoints within each plot *s* and for every pair of co‐occurring species *i* and *j*. Under the null hypothesis of spatial independence in a homogeneous environment, $n_{\mathrm{ijs}}$ follows a hypergeometric distribution

$$n_{\mathrm{ijs}}∼HG\left(N_{s},n_{\text{is}},n_{\mathrm{js}}\right)$$

where $n_{\text{is}}$ and $n_{\mathrm{js}}$ are the numbers of pinpoints occupied by *i* and *j*. A 2 *×* 2 contingency table (Appendix S1.2) summarises these counts. We performed one‐tailed Fisher exact tests for aggregation ($H_{1_{s}}^{+}$) and segregation ($H_{1_{s}}^{-}$), yielding two *p*‐values ($p_{s}^{+},p_{s}^{-}$) per pair per plot.

#### 2.2.2 Combining Local Evidence Across Plots

Local association signals may be weak individually but can become statistically robust when aggregated across multiple plots (cf. Introduction). To combine evidence, we used the Gamma Approximation of Stratified Truncated Exact test (GASTE) method (Wendling and Galiez 2025), specifically designed for discrete‐uniform *p*‐value distributions such as those derived from the hypergeometric test. We separately combined *p*‐values for local aggregations $\left\{p_{s}^{+}\right\}$ and local segregations $\left\{p_{s}^{-}\right\}$ to obtain the two combined *p*‐values ($p^{+},p^{-}$) for each species pair.

The global null hypothesis $H_{0},$ states that species *i* and *j* are spatially independent in every plot where they co‐occur. Rejection of $H_{0}$ indicates that at least one plot exhibits significant aggregation or segregation ($H_{1}^{+},H_{1}^{-}$), implying an overall significant regional association (significant regional association). We controlled the false discovery rate for all species pairs using the Benjamini–Hochberg procedure (Benjamini and Hochberg 1995) to adjust the combined *p*‐values.

To maximise the statistical power of the spatial association detection, we pre‐filtered species pairs based on whether any spatial association could be detected given their abundances. Negative associations cannot be detected for locally rare pairs, nor positive associations for locally abundant pairs. For each co‐occurring pair, we computed the minimum achievable *p*‐value based on their abundances in the plots where they co‐occurred. Only pairs with an adjusted minimum combined *p*‐value below the significance threshold (*α* = 0.05) were retained. Out of 44,002 co‐occurring pairs, we kept 3868 pairs tested for negative associations and 39,462 pairs tested for positive associations, disadvantaging positive associations in the final correction. To verify that this pre‐filtering did not artificially inflate the dominance of segregation, we reconstructed the network without this step; the relative proportions of positive and negative associations remained unchanged (Appendix S1.3).

For all species pairs with identified regional scale associations (i.e., significant adjusted combined *p*‐value), we aimed to identify the plots that contributed significantly to the regional‐scale pattern. To do so, we used the FDR‐type multiple testing procedure (Döhler et al. 2018) on the local test *p*‐values (DiscreteFDR). This procedure is suitable for discrete tests such as Fisher's test and allows the identification of plots that contributed to regional associations, thus separating mere local co‐occurrences from genuinely informative local spatial associations.

In summary, regional spatial association is determined by rejecting the overall null hypothesis that all local co‐incidences are not different from random based on a combination of *p*‐values, while local spatial association is determined post hoc, in a context of multiple tests, by rejecting the sub‐null hypotheses of random local co‐occurrence per plot (a diagram providing an overview of the workflow is available in Appendix S1.2).

### 2.3 Network Construction and Characterisation

We represented the set of significant regional species pair associations as an undirected signed regional association network with species as nodes and significant positive (aggregation) or negative (segregation) associations as links. An association between two species indicates a significant signal of spatial aggregation or segregation across all plots in which they co‐occur (cf. 2.2.2). For each species, we computed its *degree* (number of links), and for the whole network we calculated the connectance as the ratio between the observed number of association (i.e., significant signal) and the number of potential association (i.e., species co‐occurrence). Connectance was calculated for the whole signed network, as well as separately for the networks containing only positive or only negative associations. We also computed the regional clustering coefficient using igraph (Csárdi et al. 2025). The regional clustering coefficient represents the probability that two (or more) species associated with the same species are also associated with each other. In other words, it measures the local cohesiveness of a group of species (Delmas et al. 2019; Watts and Strogatz 1998). Significance of observed clustering and species degrees was assessed by comparison with null expectations generated from 999 Erdős–Rényi *G*(*n,m*) random networks with identical numbers of nodes and links (Erdos and Rényi 1960).

We identified species roles in the regional association network with an undirected binary Stochastic Block Model (SBM), computed with estimateSimpleSBM from the sbm package (Chiquet et al. 2024). The SBM partitions species into blocks (i.e., groups) that share similar association patterns (Snijders and Nowicki 1997). For each group, we measured its mean degree and within‐group connectance, defined as the ratio of the number of associations among species within the group to the total number of potential associations. We determined if the identified groups exhibited similar environmental preferences, modelling each group's abundance in relation to abiotic variables. We used Generalised Linear Models (GLMs), one per group, with a quasi‐Poisson distribution to account for overdispersion. For each group, a baseline model included total number of individuals per plot alone, while a full model also included abiotic covariates. We compared adjusted R^2^ values to evaluate the added explanatory power of abiotic variables.

### 2.4 Abiotic Influence on the Local Spatial Associations

We extracted the number of locally significant associations for each of the 141 plots with environmental data (see Section 2.2.1). We tested if the variation in the number of associations per plot could be explained by local abiotic conditions. Since the number of associations ($n_{\text{asso}}$) was likely to be correlated with, and partially explained by, the number of individuals recorded in the sampling plot, we fitted two generalised linear models (GLMs). The first model included only number of individuals as a predictor of $n_{\text{asso}}$, while the second included both the number of individuals and abiotic variables. We used a quasi‐Poisson distribution to account for overdispersion. Then, we compared the explanatory power of the two models using the adjusted R^2^, which accounts for differences in model complexity.

Highly correlated variables were removed. Mean annual precipitation was strongly correlated with FDD (Appendix S5.2) and because FDD has been shown to structure alpine plant communities (Choler 2018), we retained it. A secondary analysis using precipitation instead of FDD produced similar results (Appendix S5.3).

Stepwise selection (adjusted *R* ^*2*^ criterion) identified the most informative abiotic subset, and the relative contribution of each predictor was quantified with the hierarchical partitioning algorithm of glmm.hp (Lai et al. 2022).

### 2.5 Trait‐Based Analyses

#### 2.5.1 Group‐Level Trait Space

We first asked whether SBM groups differ in a multivariate trait space to quantify whether specific groups of species were associated with particular combinations of traits. A PCA of centred and scaled traits was performed, and group centroids were compared using a pairwise PERMANOVA test.

#### 2.5.2 CSR Strategies

We positioned every species (with complete data for SLA, LDMC and LA) in the competition–stress–ruderal (CSR) ternary diagram via the Multitrait package (He et al. 2025), following Pierce et al. (2017). We then compared mean SBM group strategies along the CSR axes using Kruskal–Wallis tests (Appendix S4.3).

#### 2.5.3 Rarity and Functional Distinctiveness

We evaluated whether the groups identified by the SBM analysis had unusual relative abundance or functional traits, computing *scarcity* (inverse of relative abundance in each plot) and functional distinctiveness as the average functional distance of a species to all the others in a given community weighted by species relative abundance (Grenié et al. 2017; Violle et al. 2017) within each plot using funrar (Grenié et al. 2017). We computed functional distinctiveness for three exemplarily traits separately (see Appendix S4.2 for the other traits) and for all five traits combined in a multivariate distance. Values were averaged per species and contrasted among SBM groups with Wilcoxon rank‐sum tests.

Spatial association inferences were conducted in Python 3.12 and statistical analyses were conducted in R 4.3. Data and scripts are archived at https://doi.org/10.5281/zenodo.19537710.

## 3 Results

Guided by our three research questions, we first described the structure of the regional network of spatial associations, then quantified how local associations depend on the abiotic context, and finally related the resulting network roles to species' functional strategies.

### 3.1 Structure of the Regional Spatial Association Network

**FIGURE 1:**
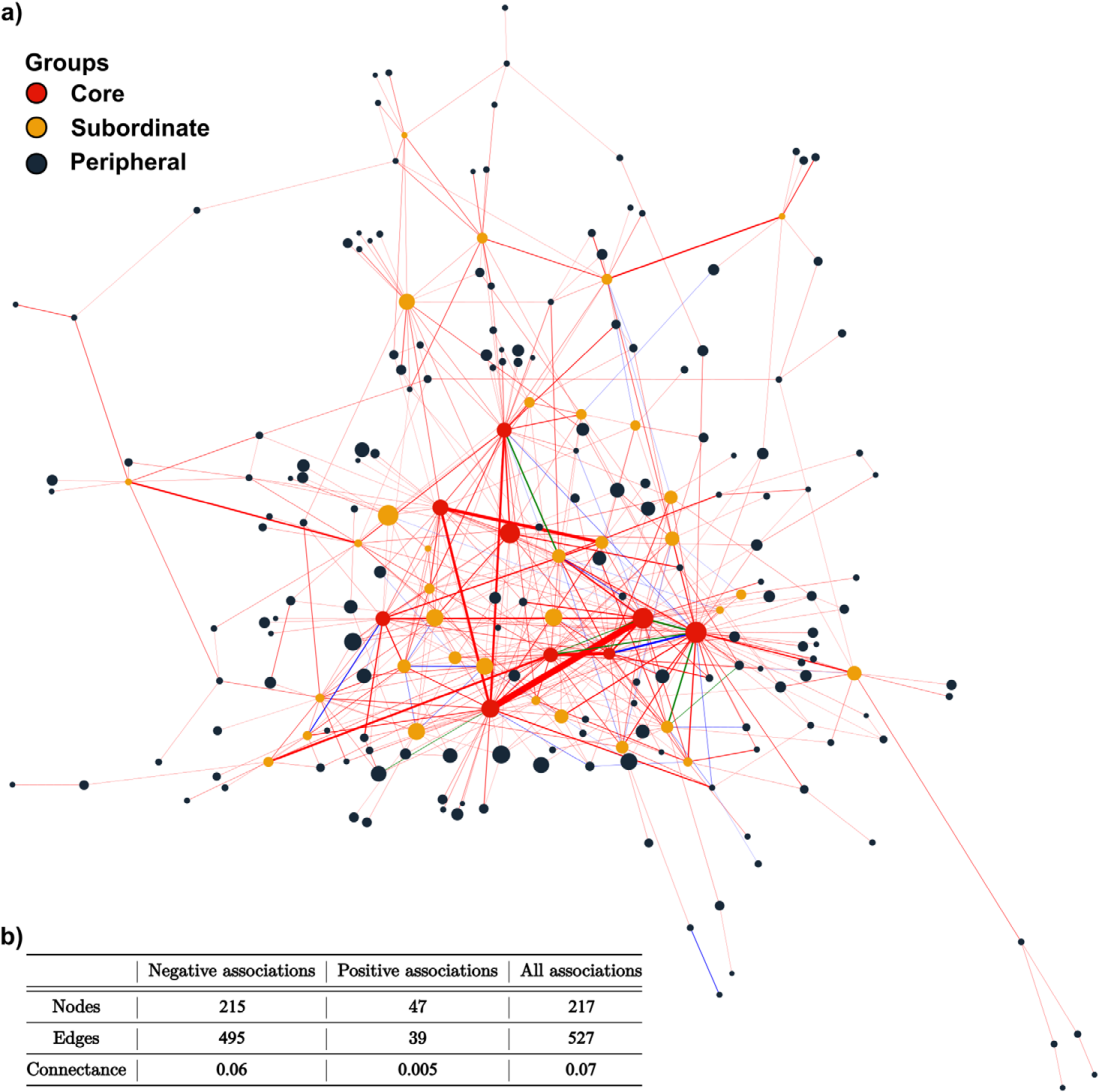
Spatial association network description. (a) Regional spatial association network, combining information from all sample plots. Red (blue) links represent negative (positive) associations, green links represent association that can be both negative or positive given the abiotic context. Nodes are coloured according to their group from the stochastic block model: Core (red), subordinate (orange) and peripheral (dark blue). (b) General description of the network.

We detected 527 significant pairwise spatial associations across the 141 plots of the 30 elevational gradients and the recorded 796 species (Figure 1a). These associations involved 217 species, 23% of the flora, and were overwhelmingly negative: 495 segregations versus only 39 aggregations, a 12.7:1 ratio (Figure 1b). Seven species pairs switched sign between plots, indicating context dependent associations.

To ensure that differences in species‐pair abundances did not introduce biases, we verified that the detection of spatial associations was not dependent on pair abundance. Our results showed that rare pairs can still display signals, whereas abundant pairs are not necessarily spatially associated (Appendix S1.2).

The regional network was extremely sparse (connectance *C* = 0.07), yet markedly more clustered than expected under an Erdős–Rényi null model (regional clustering coefficient *c* = 0.21, *p <* 0.001; Appendix S2.1). Twenty‐six species exhibited degrees significantly higher than expected under the null model (corrected *p <* 0.05; Appendix S2.2).

The Stochastic Block Model (Appendix S2.3) partitioned the 217 species into three main groups:

1. Core species (9 species): very high mean degree (32.2 ± 3) and strong within‐group connectance (75%). All nine species were highly connected (degree > null expectation, Appendix S2.1). Members include five densely tufted graminoids ( *Carex sempervirens* , *Nardus stricta* , *Avenella flexuosa* , *Festuca laevigata* and *F. violacea*) and four small shrubs ( *Juniperus communis* , *Rhododendron ferrugineum* , *Vaccinium myrtillus* and *V. uliginosum* ).
2. Subordinate species (34 species): intermediate degree (11.5 ± 0.6) with strong, mostly negative, connections to the core species (Figure 5); 13 species exceeded the null degree threshold. This group is dominated by perennial forbs and grasses (e.g., *Agrostis alpina* and *Plantago alpina*).
3. Peripheral species (174 species): few associations (2.1 ± 0.1) and minimal intra‐group connectance (0.3%). This group is mostly composed of herbaceous species (e.g., *Achillea millefolium* ), with no identifiable functional pattern.

Quasi‐Poisson GLMs (Section 2.3) modelling group abundance indicated that only the *core* group benefited from adding the abiotic predictors to a number of individuals only model, raising the adjusted *R* ^2^ from 0.24 to 0.50, driven primarily by acidic, cold sites (negative effects of pH and Growing Degree Days). In contrast, the inclusion of abiotic predictors did not significantly improve predictions of abundances for subordinate and peripheral groups (∆*R* ^2^ = 0.001 and 0.03, respectively, Appendix S2.4).

### 3.2 Abiotic Influence on Local Spatial Association Richness

The number of significant associations detected within a plot increased with soil acidity and nitrogen enrichment, when accounting for sampling intensity (number of individuals). Adding abiotic predictors to a number of individuals‐only model raised the adjusted *R* ^2^ from 0.31 to 0.46 (Table 1). Hierarchical partitioning showed that purely abiotic effects explained 40% of the variance, with soil pH alone contributing 15%. Climatic variables were comparatively minor: evapotranspiration (ETP) exerted a weak positive main effect, and GDD a weak negative effect (Table 1). Thus, soil conditions, not climate, primarily modulated the local richness of spatial associations in these alpine grasslands.

**TABLE 1:** Model predicting the number of spatial associations from local number of individuals alone versus from local number of individuals combined with abiotic factors, including interactions (e.g., Nb of individuals:ETP).

Model	Coefficients			*R* ^2^ partition	Adjusted *R* ^2^
Nb of individuals	Nb of individuals	1 × 10^*−*3^	***	100%	31%
Nb of individuals × abiotic	Nb of individuals	4 × 10^*−*3^	***	26%	46%
	pH	*−*2.3 × 10^*−*1^	***	15%	
	N%	1.4	*	13%	
	ETP	6 × 10^*−*2^	*	2%	
	Nb of individuals:ETP	*−*8.1 × 10^*−*5^	*	16%	
	Nb of individuals:N%	*−*8.9 × 10^*−4*^	n.s	19%	
	GDD	*−*1.4 × 10^*−*4^	n.s	7%	

*Note:* Significance codes: ***p < 0.001, **p < 0.01, *p < 0.05.

Abbreviation: n.s, not significant.

### 3.3 Functional Characteristics of Species Groups

**FIGURE 2:**
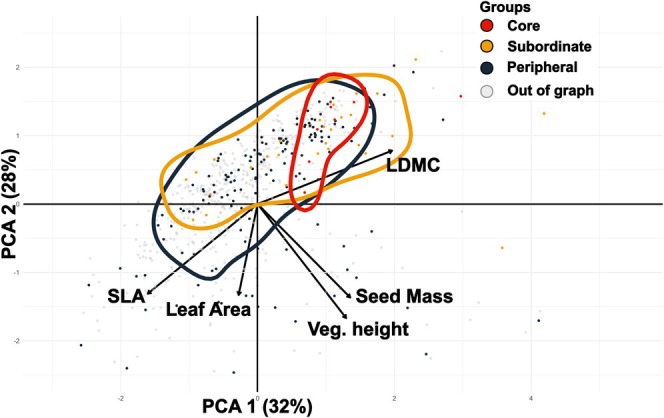
Principal Component Analysis (PCA) of the 696 plant species with trait data. Arrows indicate the direction and contribution of each trait (SLA, Leaf Area, LDMC, Seed Mass and Vegetative Height) to the first two principal components. Contour lines show the outer periphery of the trait space occupancy density of the three groups identified in the regional spatial association network: Core (red), subordinate (orange) and peripheral (dark blue). Species with no significant spatial associations are shown as grey points.

The PCA conducted over the five relevant functional traits (Figure 2) revealed a dominant SLA–LDMC axis (resource economics) and a secondary size/competition axis (leaf area, height and seed mass) explaining 60% of the total variance. While their functional spaces overlap, we observe a slight shift of the centroids of the core species toward a trait‐conservative niche with high LDMC, small leaves and short stature. Pairwise PERMANOVA indicated significant differences in trait centroids only between peripheral and core species.

**FIGURE 3:**
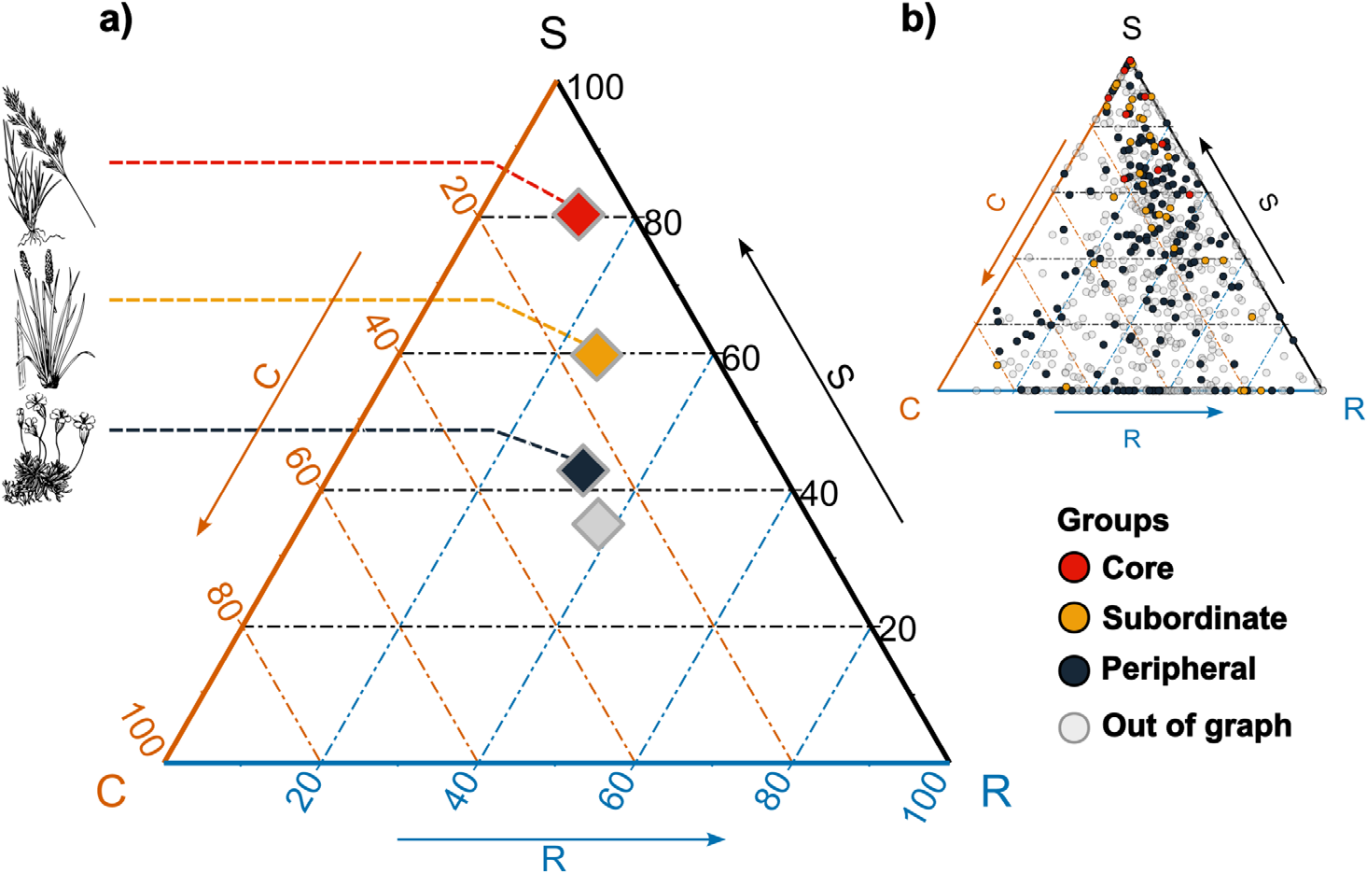
CSR strategies of species groups in the regional spatial association network. (a) Centroids of relative proportions (\%) of Competitive (C), Ruderal (R) and Stress‐tolerant (S) strategies for the three network groups: Core (red), subordinate (orange) and peripheral (dark blue). Species with no significant associations are shown in grey. Illustrations on the left depict representative species. (b) CSR strategies of all 696 species. Each point represents one species, coloured according to its group in the regional spatial association network.

Mapping species into the Competitor, Stress‐tolerant, Ruderal (CSR) trait space confirmed this observation (Figure 3). Core and subordinate species clustered toward the stress‐tolerant apex (centroid of the core group in the C:S:R space = 7:80:13%), whereas peripheral and non‐associated species spanned the entire triangle (Figure 3b).

**FIGURE 4:**
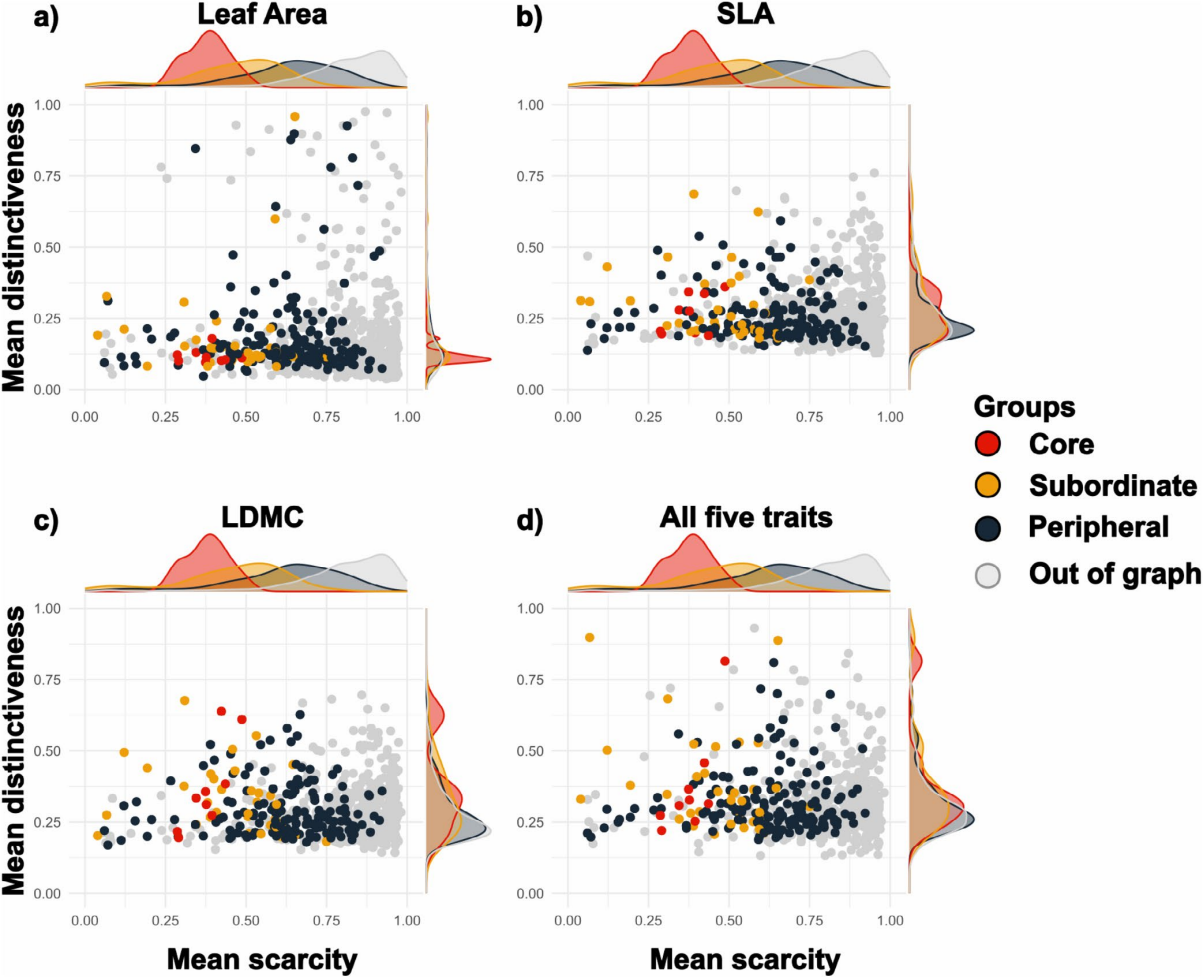
Biplots of species' mean functional distinctiveness against mean scarcity. Panels (a) to (c) display trait‐specific distinctiveness for (a) Leaf Area, (b) SLA and (c) LDMC. Panel (d) shows distinctiveness considering all five traits (Leaf Area, SLA, LDMC, Vegetative height and Seed mass) combined with a multivariate distance. Species are coloured according to their group in the regional spatial association network: Core (red), subordinate (orange), peripheral (dark blue) and non‐associated species (grey). Marginal distributions are indicated on the sides. For figure readability the results for Vegetative height and Seed mass are displayed in Appendix S4.2.

Core species were locally abundant (lower scarcity; Wilcoxon *p <* 0.001; Figure 4) but not functionally distinct from the other two species groups. LDMC distinctiveness was only marginally higher (Figure 4c) and multivariate distinctiveness did not differ from other groups (Figure 4d). Two evergreen shrubs, *R. ferrugineum* and *J. communis* , were the exceptions, with highly distinctive values for LDMC.

## 4 Discussion

In this paper, we disentangled plot‐level co‐occurrence from local *spatial associations* in mountain plant communities. Our results reveal three key insights: (i) the regional network is dominated by negative associations between a small number of core structuring species; (ii) these core species share conservative leaf traits and tend to be stress‐tolerant and (iii) local associations are primarily driven by soil properties (increase with acidity and nitrogen) rather than thermal gradients.

### 4.1 A Segregation‐Dominated Network Structured by Stress Tolerant Species

Negative associations outnumbered positive ones, aligning with expectations for species‐rich, densely vegetated alpine meadows. Facilitation is often emphasised in stressful environments (Callaway et al. 2002; Choler et al. 2001; Michalet et al. 2015), for example through nursing effects (Anthelme et al. 2014; Olofsson 2004; Reid et al. 2010) or buffering effects (e.g., wind protection, Callaway et al. 2002; Choler et al. 2001) in sparsely vegetated systems. In densely vegetated plots, potential facilitative interactions are likely diluted among many neighbours, and difficult to detect. In contrast, competitive interactions are known to play a key role in dense vegetation (Bektaş et al. 2023; Huelber et al. 2011). The observed dominance of segregation therefore likely reflects a combination of potential competition and facilitative processes, with the former being strong and the latter weak, diffuse and hard to detect at the pairwise level.

The network topology was highly clustered and centralised, with nine *core* species, primarily highly abundant tussocks and acidophilic shrubs, acting as structural hubs. These core species modulate the overall structure of the regional network of spatial associations. Subordinate species are spatially organised around them, displaying high intra‐ and inter‐group connectivity consistent with hierarchical community patterns reported earlier (Losapio et al. 2018). Peripheral species, meanwhile, appear spatially constrained by both core and subordinate species, effectively filling gaps in community structure.

Core species were slightly more conservative than the other species (high LDMC, small leaves, short stature), and tended toward the stress‐tolerant (S) apex of the CSR strategy triangle (Pierce et al. 2013). This dominance of stress‐tolerant rather than competitive, acquisitive species may seem paradoxical given the prevalence of negative associations but core species also showed high relative abundance and were thus strongly influenced by environmental filtering (Arnillas and Cadotte 2019; Chalmandrier et al. 2017), known to select for conservative traits in mountains (Bektaş et al. 2023). In congruence with this idea, we found that core species were functionally common within their plots. Their structural role appears to arise from abundance and wide environmental breadth, not from rare trait combinations—echoing the idea that common traits, if coupled with high abundance, can steer community structure (Munoz et al. 2023).

Interestingly, most species (*∼* 73%) did not exhibit any spatial association, likely reflecting weak interactions. These species may compartmentalise resource use (Ashton et al. 2010), reducing niche overlap and enhancing coexistence (Chesson 2000). Once environmental filters are passed, random spatial mixing may be the norm rather than the exception in these communities. Overall, our results reveal a hierarchical structure (core–subordinate–peripheral) forming a structural backbone within a ‘matrix’ of randomly distributed species.

### 4.2 Soils, More Than Climate, Drive Local Associations

Contrary to many alpine studies emphasising temperature as the main driver of species distributions (e.g., Choler 2018), we found that soil variables, specifically pH and nitrogen content, were the key drivers of local spatial associations. The positive effect of nitrogen aligns with resource‐competition theory: in more productive sites, dense stands and competition for nutrients intensify either spatial segregation (root foraging and shoot shading) or tight clustering around nutrient hotspots (Tilman 1984). Although slightly acidic soils have historically been considered favourable, recent work questions a general link between pH and soil fertility (Hartemink and Barrow 2023); accordingly, we found no relationship between soil nitrogen and pH (Appendix S5.2). The negative effect of pH mirrors the environmental preferences of several core acidophilic species (*Vaccinium*, *Rhododendron*, *Juniperus*; Landolt et al. 2010), which acidify soils through their litter (Körner 2003), reinforcing fine‐scale spatial structuring. These results suggest that community composition (presence of dominant shrubs) feeds back into network structure not only by direct biotic control but also by indirect plant–soil functioning‐feedbacks (van der Putten et al. 2013).

Interestingly, we identified seven species pairs whose spatial association signs shifted between plots. Although statistical power was insufficient to pinpoint drivers, site‐level analyses revealed that negative associations were more frequent in acidic, high carbon to nitrogen ratio soils (Appendix S3). This pattern aligns with experimental evidence of interaction switches from competition to facilitation (Bimler et al. 2024) and supports the view that network structure varies across environmental gradients (Pellissier et al. 2018).

### 4.3 Synthesis and Implications

Overall, our results portray mountain open vegetation as communities that are spatially structured around a handful of widespread, stress‐tolerant species that exert disproportionate influence through segregation processes, particularly in fertile, acidic soils. These core species, together with a set of subordinate species, form a spatial structural backbone around which the many other co‐occurring species seem to underly neutral dynamics.

This structure has critical implications for how mountain communities may reorganise under rapid climate change (Chan et al. 2024), and increasing species richness at higher elevations (Steinbauer et al. 2018). The way newly arriving species either integrate into this existing backbone or restructure it may determine whether high‐elevation communities converge toward lower‐elevation assemblages (Savage and Vellend 2015) or follow alternative trajectories (Staples et al. 2022).

### 4.4 Limitations and Future Directions

Our study strives to infer biotic processes from spatial patterns in plant communities, an approach with known limitations and potential for spurious associations (Blanchet et al. 2020). By focusing on intra‐plot spatial structure rather than broad‐scale co‐occurrence, we aimed to minimise confounding effects of environmental filtering and dispersal limitation. However, even though plots were selected to ensure homogeneous slope, aspect and vegetation cover (Thuiller et al. 2024), fine‐scale environmental heterogeneity within 30 m reading lines, such as microtopography or fine‐scale soil variations, may still generate spatial patterns that reflect micro‐environmental filtering rather than species interactions (Conti et al. 2017).

The regional network contained only 217 nodes and 7% of potential associations, despite over 90,000 pinpoint observations. The scarcity of positive associations may reflect abundance constraints, since it was not possible to statistically detect any signal of positive association for rare species pairs (see Section 2.2.2). Detecting rare but ecologically important interactions therefore remains challenging. Our presence–absence data represent a very fine scale sampling, likely missing interactions occurring at slightly larger spatial scales of centimetres in the tens range (e.g., nursing). Extending the framework to quantitative cover data could improve sensitivity, as abundance covariation can capture facilitative or competitive processes (Losapio et al. 2021). However, the challenge to decide which spatial scale is the right one to detect species coexistence would remain.

In our functional analysis, we had no information on root traits which could provide valuable insights into belowground biotic interactions (Semchenko et al. 2018). However, these traits are difficult to measure, or to find in existing databases (e.g., Guerrero‐Ramírez et al. 2021; Kattge et al. 2020). Similarly, because our study focused on the lower vegetation layer, we excluded the few juvenile trees that were recorded (*∼* 5% of the data) since we only had access to the adult traits.

Finally, niche theory predicts greater competition among species with high niche overlap (Macarthur and Levins 1967). Future work could predict spatial associations directly from trait distances (Chalmandrier et al. 2021), an approach successful in food‐web ecology (Bartomeus et al. 2016; Wootton et al. 2024), offering a mechanistic link between species traits and community structure.

### 4.5 Take Home Message

**FIGURE 5:**
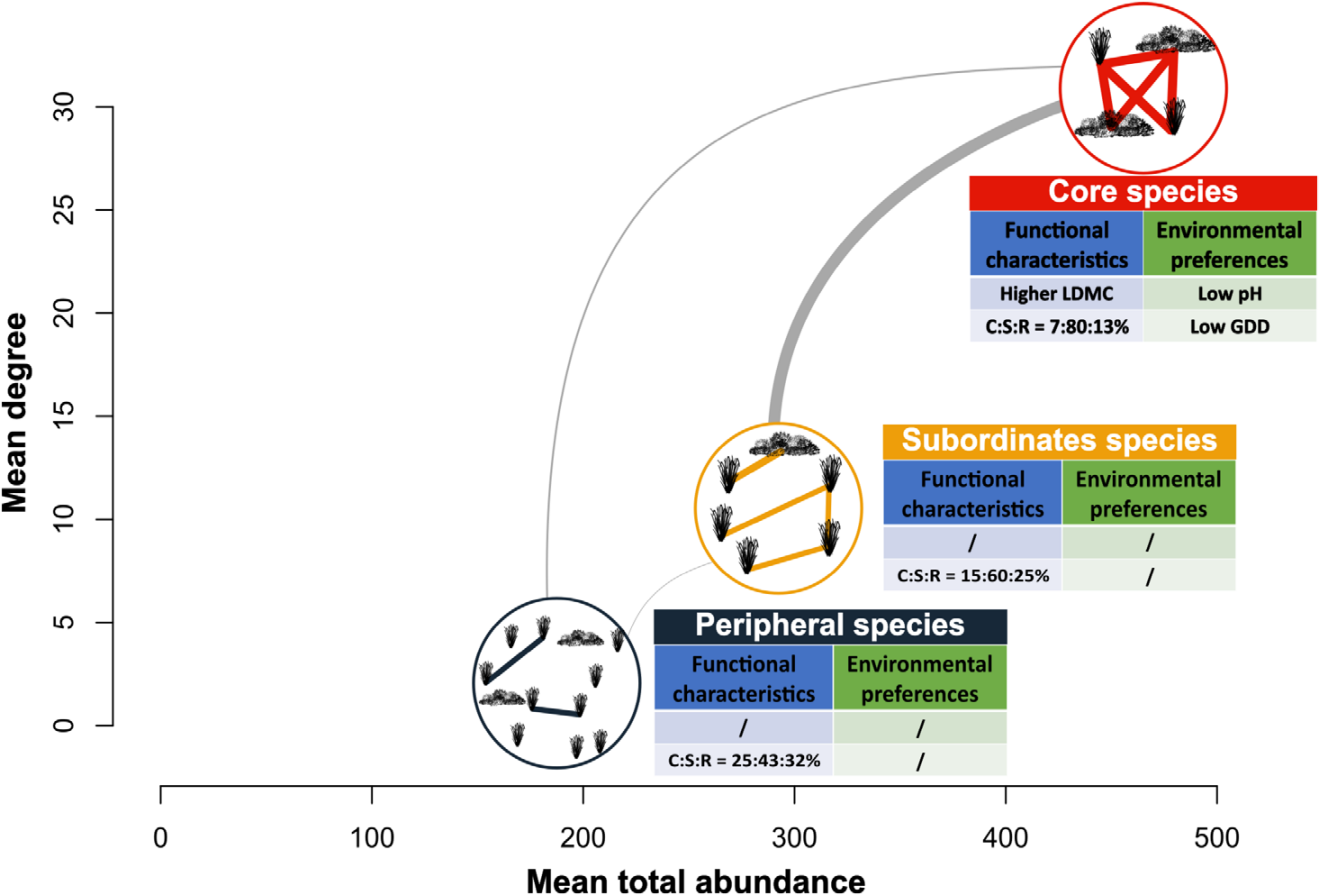
Summary of the results: The three groups of species: Core, subordinate and peripheral; are positioned according to the mean total abundance and mean degree of the nodes that comprise them. Each group is characterised by its dominant ecological strategy (C:S:R ratio), functional traits and abiotic preferences. Internal links represent intra‐group connectance, while the thickness of grey links between groups indicates inter‐group connectivity.

In summary, our multi‐scale, trait‐informed network analysis reveals that alpine plant communities are mostly structured by segregation around stress‐tolerant dominants (Figure 5), with soil chemistry, not climate, being the main abiotic driver at local scale. Recognising the dual role of dominant species, as abundant biomass and as network hubs, offers a powerful lens through which to anticipate how alpine ecosystems will reconfigure under ongoing environmental change.

## Author Contributions

M.R., T.M. and W.T. originally designed the study, A.W., T.M., D.G., C.G. and W.T. contributed to the final conception of the study. A.W. and C.G. developed the statistical framework for detecting spatial associations. M.R. and A.W. performed the network analysis. J.R. managed the database. People from O.C. collected the data. M.R. wrote the first draft of the manuscript together with T.M. and W.T. All authors contributed to the revisions.

## Funding

This work was supported by Agence Nationale de la Recherche, DYNABIOD (ANR‐25‐APDY‐0001), EcoNet (ANR‐18‐CE02‐0010‐01), GlobNet (ANR‐16‐CE02‐0009), PEG2 (ANR‐22CE45‐0033), Reframe (ANR‐24‐CE02‐7222), TransAlps (ANR‐20‐CE02‐0021); Office Français de la Biodiversité, Sentinelles des Alpes 2020–2022, Sentinelles des Alpes 2023, Sentinelles des Alpes 2024; Analyses et Expérimentations pour les Ecosystèmes, ANR‐11‐INBS‐0001AnaEE‐Services.

## Supporting information


**Appendix S1:** Inferring spatial association.
**Appendix S2:** Structure of the regional spatial association network.
**Appendix S3:** Context dependency of spatial associations.
**Appendix S4:** Functional analysis.
**Appendix S5:** Environmental covariates.
**Appendix S6:** Consortium ORCHAMP.

## Data Availability

The data and code that support the findings of this study are openly available at https://doi.org/10.5281/zenodo.19537710.
