# Supplementary material for: Beyond Co‐Occurrence: Multi‐Scale Evidence for Segregation‐Dominated Plant Networks in the French Alps
Source: Ecol Lett. 2026 May 1;29:e70393. doi: 10.1111/ele.70393 (PMC13135133; doi:10.1111/ele.70393)
Supplement: Supplementary file 1 — Appendix S1: Inferring spatial association. Appendix S2: Structure of the regional spatial association network. Appendix S3: Context dependency of spatial associations. Appendix S4: Functional analysis. Appendix S5: Environmental covariates. Appendix S6: Consortium ORCHAMP. [file ELE-29-0-s001.pdf]

# Supporting Informations for: Beyond Co-Occurrence: Multi-Scale Evidence For Segregation-Dominated Plant Networks In The French Alps

## Appendix S1: Inferring spatial association

### S1.1: Summary of the analysis scales

**Table 1:** Summary of the spatial scales analysed and their ecological interpretation.

| | Spatial scale | Ecological meaning |
|---|---|---|
| Co-location | Pinpoints (intra-plot) | Indicates the potential for fine-scale coexistence |
| Local spatial association | Plot | Species pairs exhibit locally a non-random spatial distribution |
| Co-occurrence | Plot | Species pairs occur in the same plot without a particular spatial pattern |
| Regional spatial association | All plots ($\sim$ French Alps) | Significant association detected across all co-occurring plots (combination of plot-level $p$-values) |
| Regional association network | All plots ($\sim$ French Alps) | Network integrating all significant regional spatial associations |

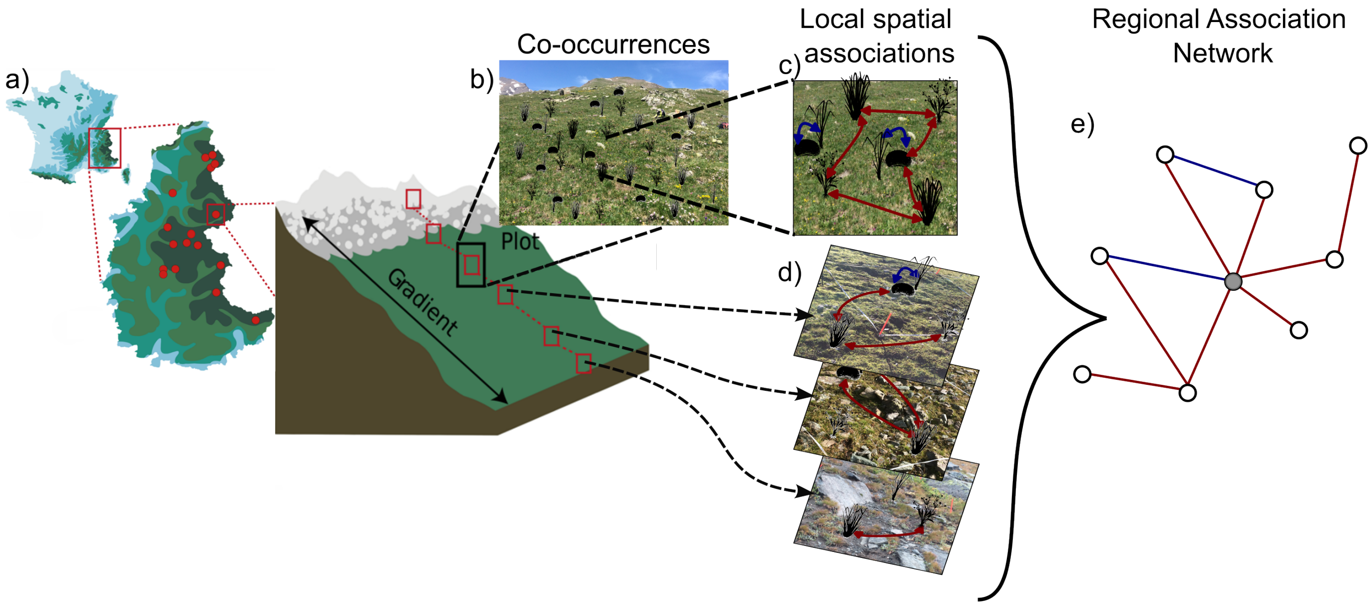


**Figure 1:** Conceptual representation of the key concepts developed in the main text. (a) Map of the ORCHAMP monitoring sites across the French Alps. (b) *Co-occurrence*: within a given ORCHAMP plot, all plant species present are considered to co-occur. (c) *Local spatial association*: among these co-occurring species, some pairs exhibit a non-random spatial distribution (positive or negative association). (d) The spatial structure is analysed for all the ORCHAMP plot. Different plots may exhibit variation in their spatial structure. (e) *Regional association network*: all significant spatial associations detected across plots are aggregated into a single signed network (positive associations in blue, negative in red), which may display emergent structural properties—for example, high degree centralisation around the grey node.

## S1.2: Details on methods

Within each plot $s$ and for every pair of co-occurring species $i$ and $j$, we tested whether the observed number of shared pinpoints ($n_{ijs}$) differed from a random allocation of individuals to the $N_s = 300$ pinpoints. A $2 \times 2$ contingency table summarises these counts (Table 2).

**Table 2:** 2x2 contingency table for plot $s$

| | Species $i$ | | |
|---|---|---|---|
| Species $j$ | Presence | Absence | Total |
| Presence | nb co-locations ($\boldsymbol{n_{ij_s}}$) | nb $j$ alone | total $j$ ($\boldsymbol{n_{j_s}}$) |
| Absence | nb $i$ alone | nb pins without $i$ & $j$ | total pins without $j$ |
| Total | total $i$ ($\boldsymbol{n_{i_s}}$) | total pins without $i$ | total pins ($\boldsymbol{N_s}$) |

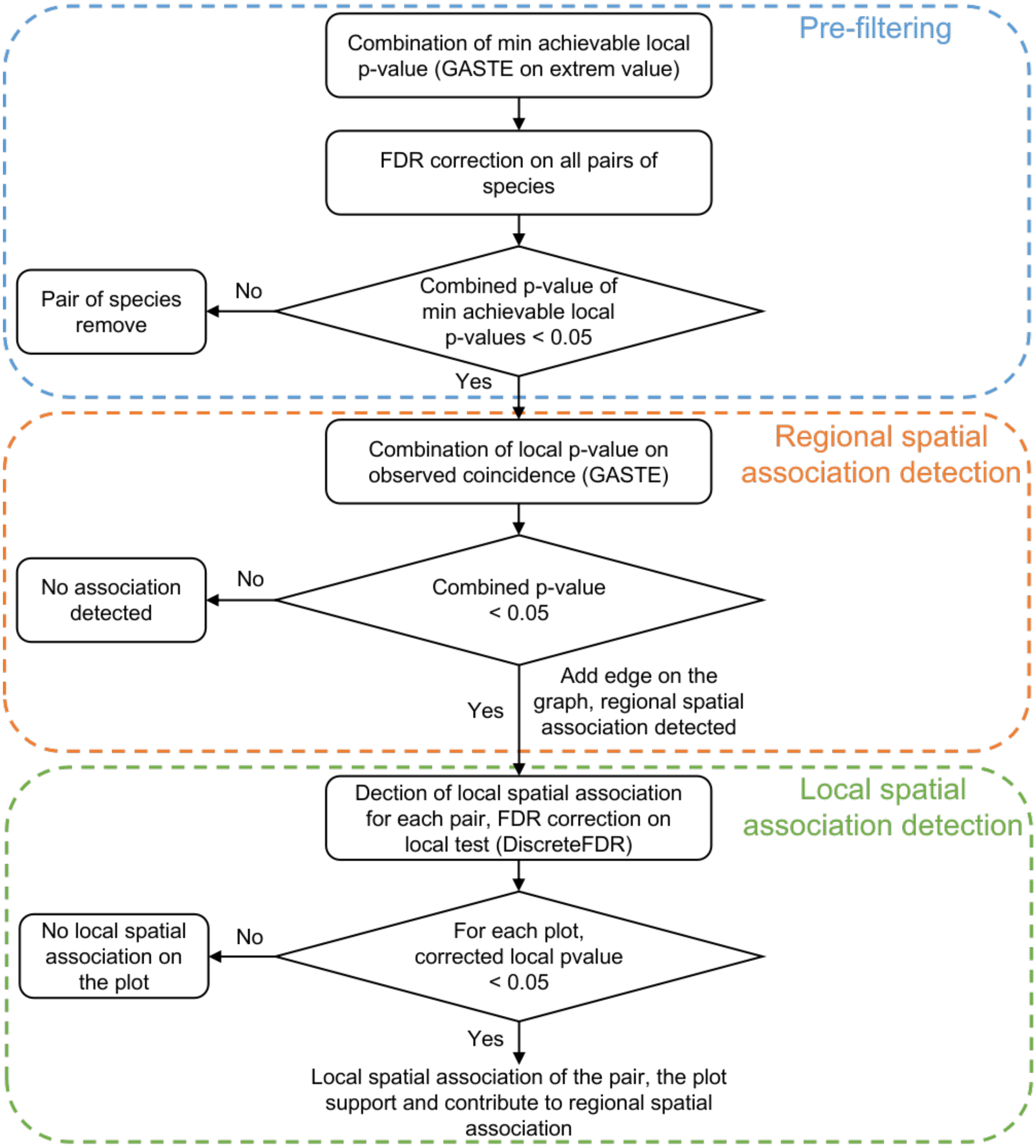


**Figure 2:** Diagram of an overview of the method, first part is the pre-filtering, second part is the regional spatial association detection, and the third part is the local spatial association detection

To assess potential biases arising from species abundance in the detection of spatial associations, we

computed for each species pair the minimum achievable combined $p$-values (Figure 2). These minimum $p$-values were then plotted against the sum of the average abundances of the two species across all plots where they co-occurred (Figure 3). When highlighting the species pairs that are actually part of the spatial association network (i.e. with realized combined $p$-values $< 0.05$), we observed that they are uniformly distributed: rare species pairs can display signals of spatial association, while conversely, abundant species pairs are not necessarily spatially associated. Even among the most abundant pairs, we observed strong disparities in realized spatial associations despite their strong potential signal (i.e. low minimum achievable $p$-values; Figure 4).

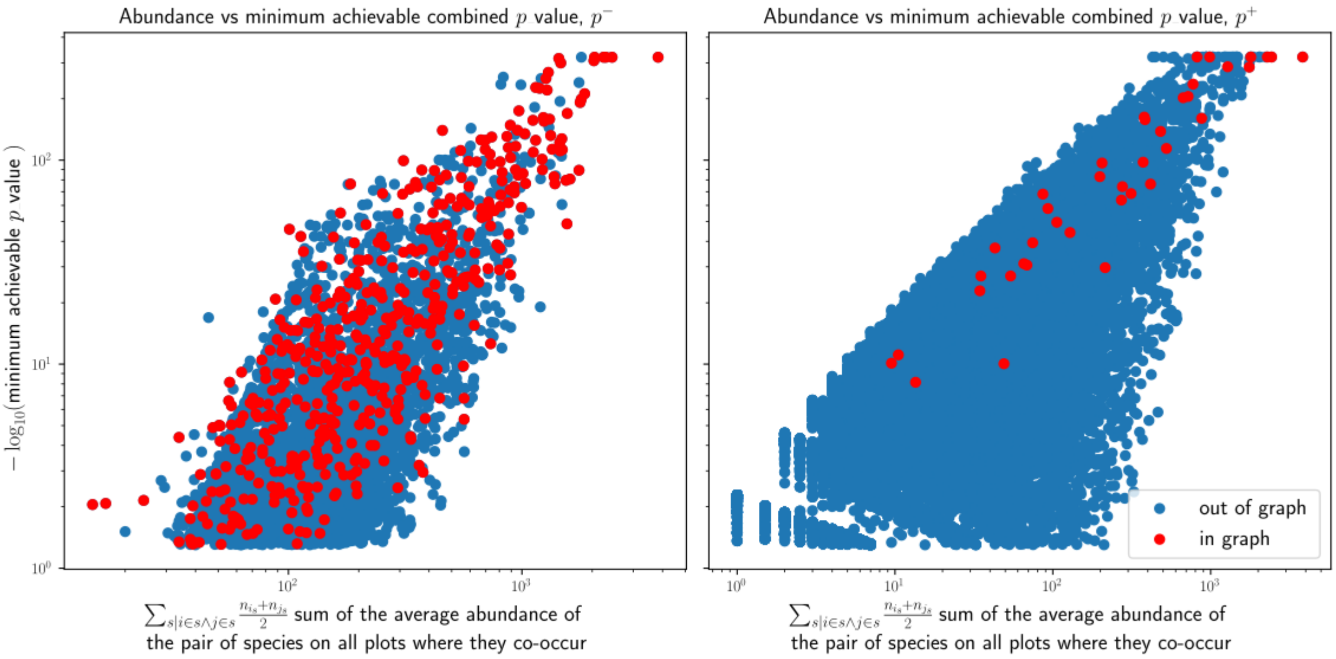


**Figure 3:** Minimum achievable combined $p$-values for negative associations (left panel) and positive associations (right panel) ($-\log_{10}$-transformed values) plotted against the summed average abundances of each species pair. Red dots indicate species pairs that are part of the spatial association network.

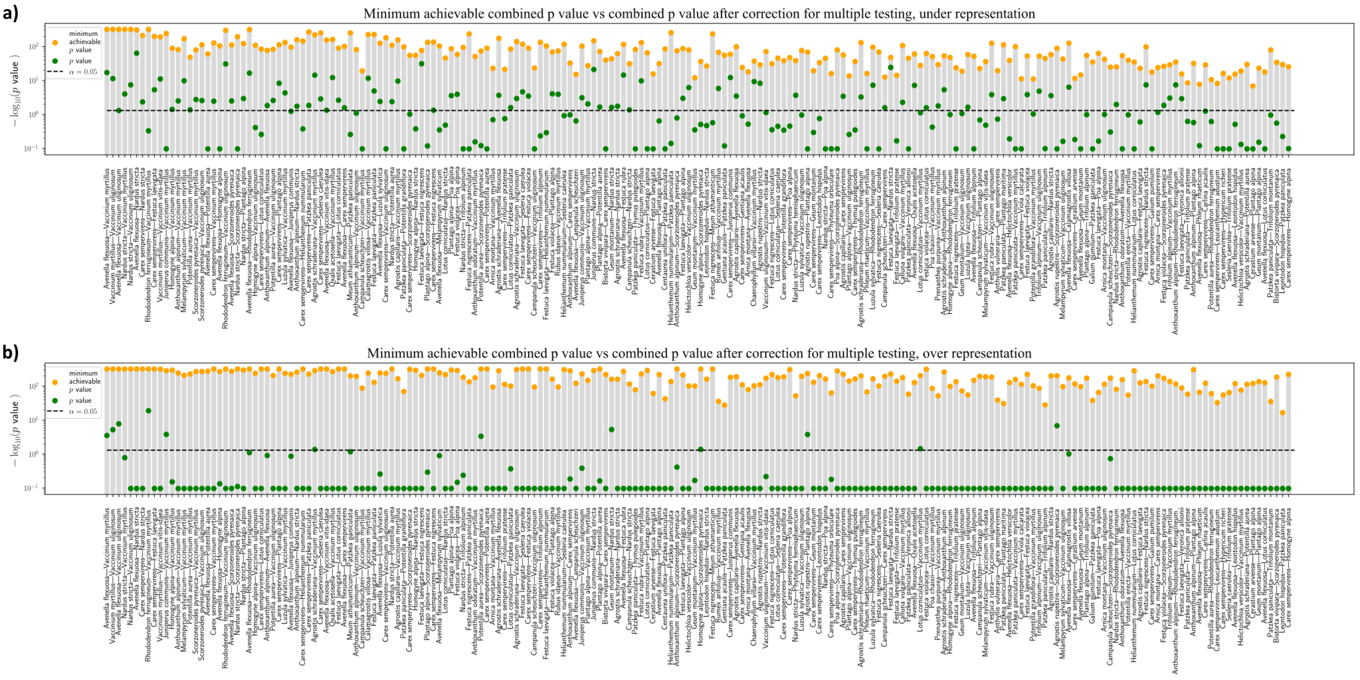


**Figure 4:** Minimum achievable combined *p*-values (yellow dots) and observed combined *p*-values (green dots), both $-\log_{10}$-transformed, for readability, only the 100 most abundant species pairs are shown. (a) Negative associations and (b) positive associations.

## S1.3: Consequences of the pre filtering

Because negative (positive) associations cannot be detected for pairs of locally rare (abundant) species, we first computed, for each co-occurring species pair, the minimum achievable p-value given their abundances across the plots where they co-occurred. Only pairs with an adjusted minimum p-value below the significance threshold ($\alpha = 0.05$) were retained (Figure 2). Out of 44,002 co-occurring pairs, this filtering step kept 3,868 pairs for testing negative associations and 39,462 pairs for testing positive associations, creating a strong imbalance (1:10.2) that could disadvantage positive associations in the final correction procedure.

To evaluate whether this imbalance could bias our results, we built different spatial association networks. One without applying this pre-filtering step, thus testing all 44,002 species pairs for both positive and negative associations. And two others where the same number of pairs were tested, respectively, for negative and positive associations. In these two cases, in order to match the number of pairs, the first n lowest minimum achievable p-values are retained. The main characteristics of these four networks (with and without pre-filtering) are presented in Table 3. The unfiltered network showed a substantial reduction in the number of negative links, yet negative associations remained strongly dominant (86%). In the cases where the same pre-filtering restriction to positive, respectively negative, association is applied, we found 495 significant negative and 79 significant positive associations, respectively 244 significant negative and 39 significant positive associations. In these two cases, negative links represent a constant 86% of the network, which is similar to our previous test without the pre-filtering step. Therefore, the prevalence of negative associations in our main results is unlikely to be an artefact of the pre-filtering procedure.

**Table 3:** Spatial association network comparison with and without the pre-filtering procedure

| | Neg tested | Pos tested | Negative | Positive | Both | Order | Size | % Negative |
|---|---|---|---|---|---|---|---|---|
| No filter | 44002 | 44002 | 241 | 39 | 4 | 138 | 276 | 86% |
| Same nb tested pos | 39462 | 39462 | 244 | 39 | 4 | 139 | 279 | 86% |
| Pre-filter | 3868 | 39462 | 495 | 39 | 7 | 217 | 527 | 93% |
| Same nb tested neg | 3868 | 3868 | 495 | 79 | 13 | 226 | 561 | 86% |

Since only 48% of the negative links remained in the network constructed without pre-filtering, we examined which types of nodes were affected by comparing the presence of species from each broad group (*core*, *subordinate*, *peripheral*) across the two networks (Table 4). The only species missing in the unfiltered network were those classified as peripheral in the pre-filtered network. This indicates that the pre-filtering step increases statistical power and allows the detection of more cryptic associations, without altering our overall conclusions.

**Table 4:** Role of the nodes affected by the pre-filtering procedure

| | Core | Subordinates | Peripheral |
|---|---|---|---|
| No filter | 9 | 34 | 95 |
| Pre-filter | 9 | 34 | 174 |

# Appendix S2: Structure of the regional spatial association network

We computed the *regional clustering coefficient* using `igraph` (Csárdi et al., 2025). The regional clustering coefficient represents the probability that two (or more) species associated with the same species are also associated with each other. In other words, it measures the local cohesiveness of a group of species (Delmas et al., 2019; Watts and Strogatz, 1998). Significance of observed clustering and species degrees was assessed by comparison with null expectations generated from 999 Erdős–Rényi $G(n, m)$ random networks with identical numbers of nodes and links (Erdos et al., 1960).

## S2.1: Network clustering

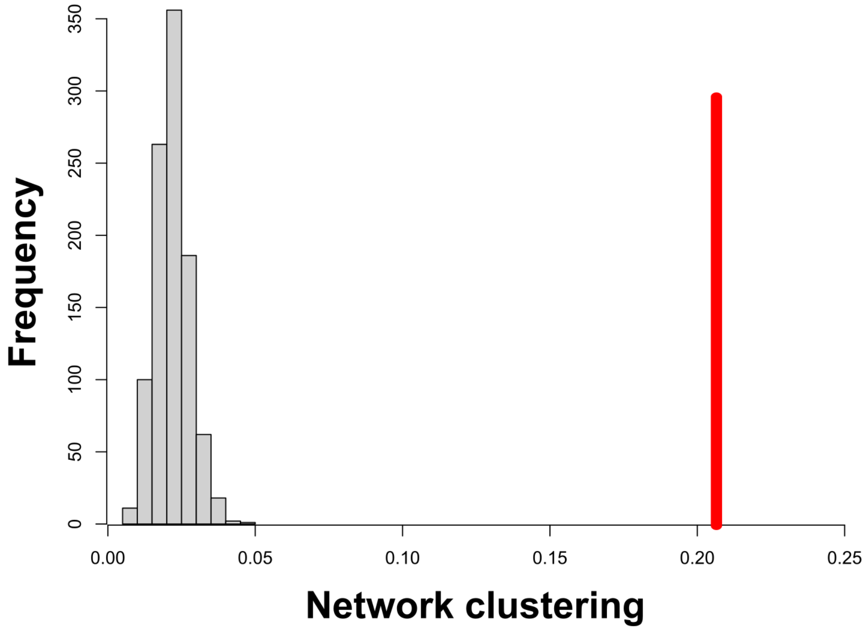


**Figure 5:** Observed measure of clustering (in red) and the distribution of the clustering measure of 999 random networks (grey histogram).

The regional network was markedly more clustered than expected under an Erdős–Rényi null model (regional clustering coefficient $c = 0.21$, $p < 0.001$).

## S2.2: Species connection

For each species, we computed its *degree* (number of links, $k$). We then compared the observed species degree to a random expectation derived from 999 Erdős–Rényi model (Erdos et al., 1960) $G(n, m)$ random graphs with the same number of nodes and links. $P$-values were calculated as the probability for the observed number of the degree to be higher (or lower) than in the random networks (ie. the proportion of random observations lower (or higher) than the observed values), and were subsequently FDR-corrected (Benjamini and Hochberg, 1995). Species degrees were standardized using Z-scores as follows:

$$Z(k) = (k_{\text{obs}} - k_{\text{rnd}})/\text{sd}(k_{\text{rnd}})$$

Twenty-two species showed significantly higher degrees than expected by chance, including all nine core species and thirteen subordinate species (Figure 6).

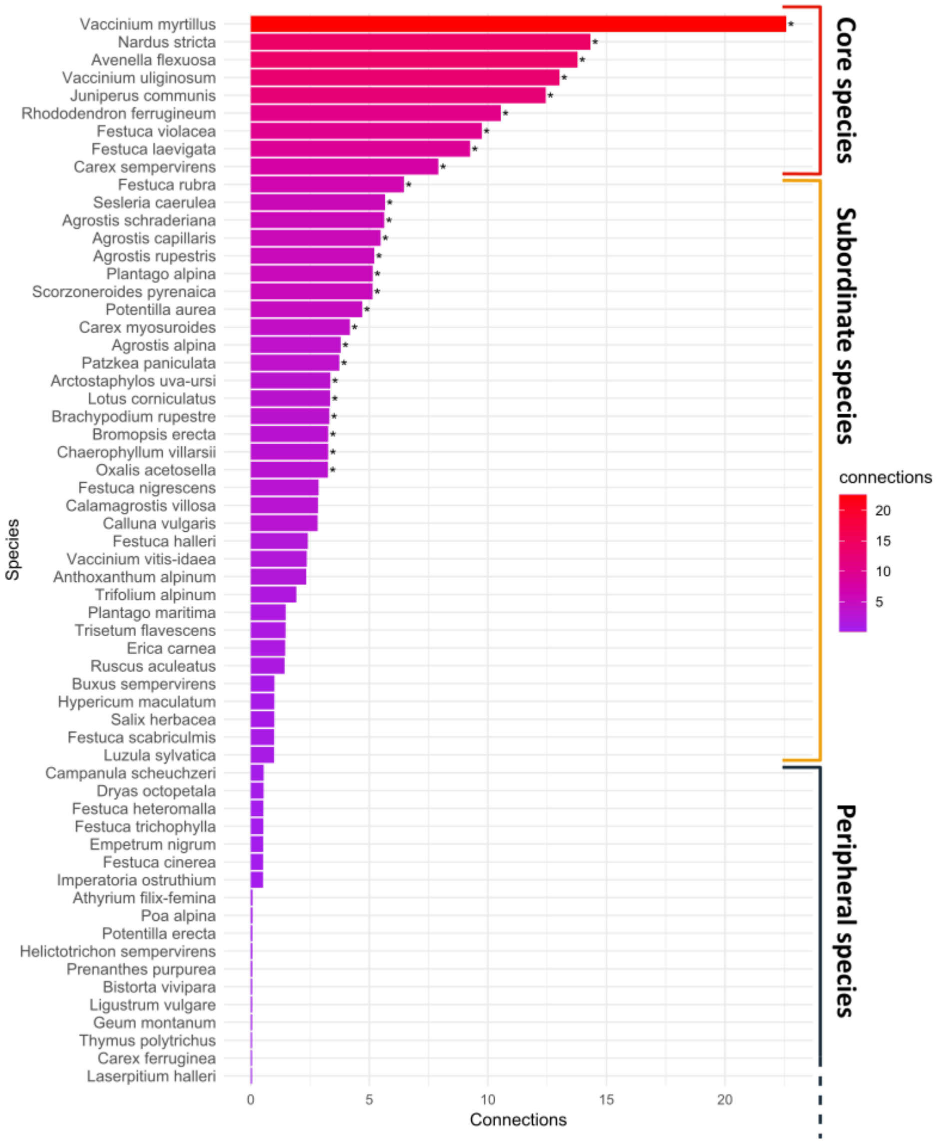


**Figure 6:** Z-scores of species degrees in the spatial association network. Bars show standardized species-level degrees. Asterisks mark species with significantly higher degrees than expected under a null model (FDR-corrected $p < 0.05$). For readability, only positive Z-scores are shown.

## S2.3: Stochastic Block Model

We identified species roles in the regional association network with an undirected binary Stochastic Block Model (SBM), computed with estimateSimpleSBM from the sbm package (Chiquet et al., 2024). The SBM partitions species into blocks (i.e. groups) that share similar association patterns (Snijders and Nowicki, 1997)

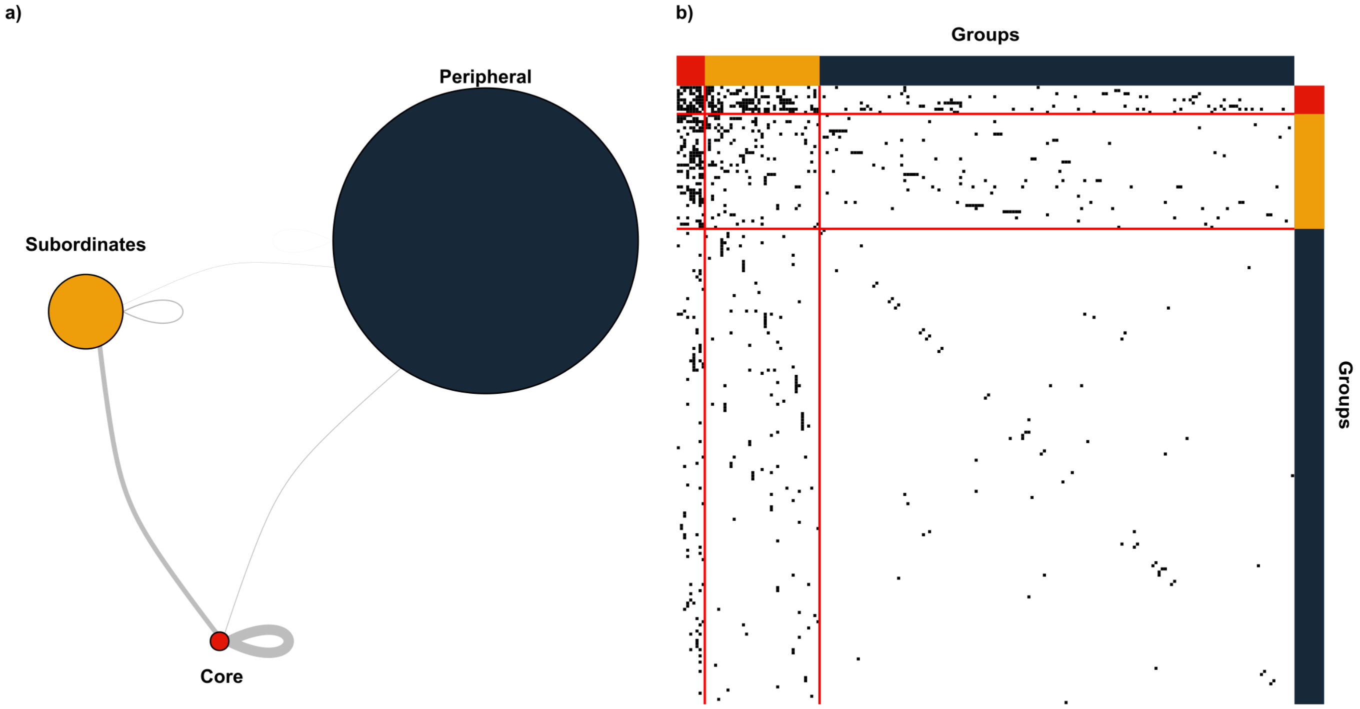


**Figure 7:** Structure of the species association network based on the Stochastic Block Model (SBM). a) Schematic representation of the three groups identified by the SBM. Node size is proportional to the number of species in each group, and edge thickness reflects the density of associations (intra- or inter-group). b) Reordered adjacency matrix showing the distribution of associations according to group membership. Red lines delimit groups.

## S2.4: Abiotic influence on the species groups

**Table 5:** Summary statistics for species groups identified in the spatial association network using the Stochastic Block Model. Number of species per group, mean degree (± standard error), intra-group connectance (proportion of realized associations within each group), and the increase in adjusted $R^2$ ($\Delta R^2$) when abiotic predictors are added to an number of individuals-only model.

| Groups | Number of species | Mean degree | Intra-Group Connectance | $\Delta R^2$ |
|---|---|---|---|---|
| Core | 9 | $32.2 \pm 3$ | 75% | 0.26 |
| Subordinate | 34 | $11.5 \pm 0.6$ | 15% | 0.001 |
| Peripheral | 174 | $2.1 \pm 0.1$ | 0.3% | 0.01 |

# Appendix S3: Context dependency of spatial associations

The spatial association network revealed that seven species pairs switched association sign between plots, indicating context-dependent interactions. Because we lacked the statistical power to formally test how abiotic conditions influenced the sign of each individual association, we instead examined more broadly which sites provided evidence for: (i) positive associations, (ii) negative associations, (iii) both, or (iv) no detected association for each switching pair. We identified these site categories using the same procedure described in the main text (Section 2.2.2) and Appendix S1.1.

Across all species pairs that switched sign, we found 16 sites supporting only negative associations, 9 supporting only positive associations, 8 supporting both positive and negative associations, and 28 sites where the species only co-occurred without a significant association.

To explore whether these site categories corresponded to distinct environmental conditions, we performed a PCA on site-level environmental variables and projected the four site categories (negative, positive, both, none) onto the resulting ordination (Figure 8).

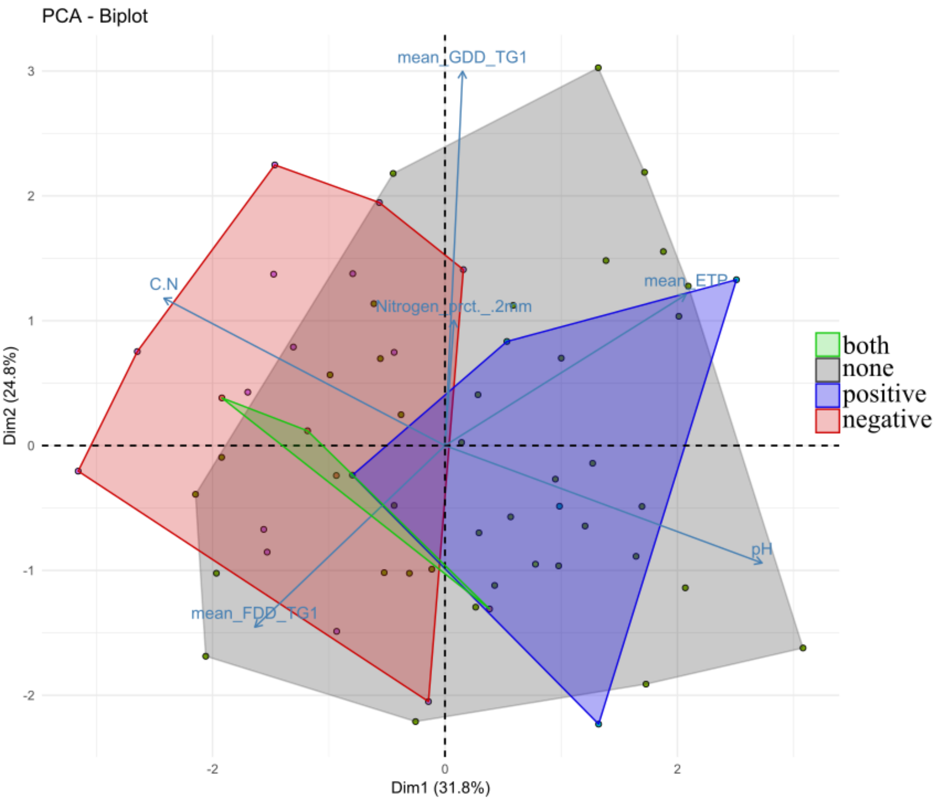


**Figure 8:** Principal Component Analysis (PCA) of the 61 sites where the species pairs that switched association sign were found to co-occur at least once. Coloured polygons represent the convex hulls enclosing sites belonging to each category (negative, positive, both, or none).

A clear gradient from negative to positive spatial association appear on the PCA, suggesting that negative association appear mainly in site with low pH and high level of C:N ratio.

# Appendix S4: Functional analysis

## S4.1: Gap-filling sensitivity analysis

To perform the functional analysis in the main text we used a gap-filling method to complete our functional data. To verify if this traits imputation introduce bias in our functional analysis we perform a second analysis, considering only the species for which we had complete measured trait data.

Out of the 693 species analysed in the main text we keep the 422 species with complete measured data and perform the PCA on function traits (Figure 9), the functional strategies CSR (Figure 10) and the functional rarity analysis (Figure 11).

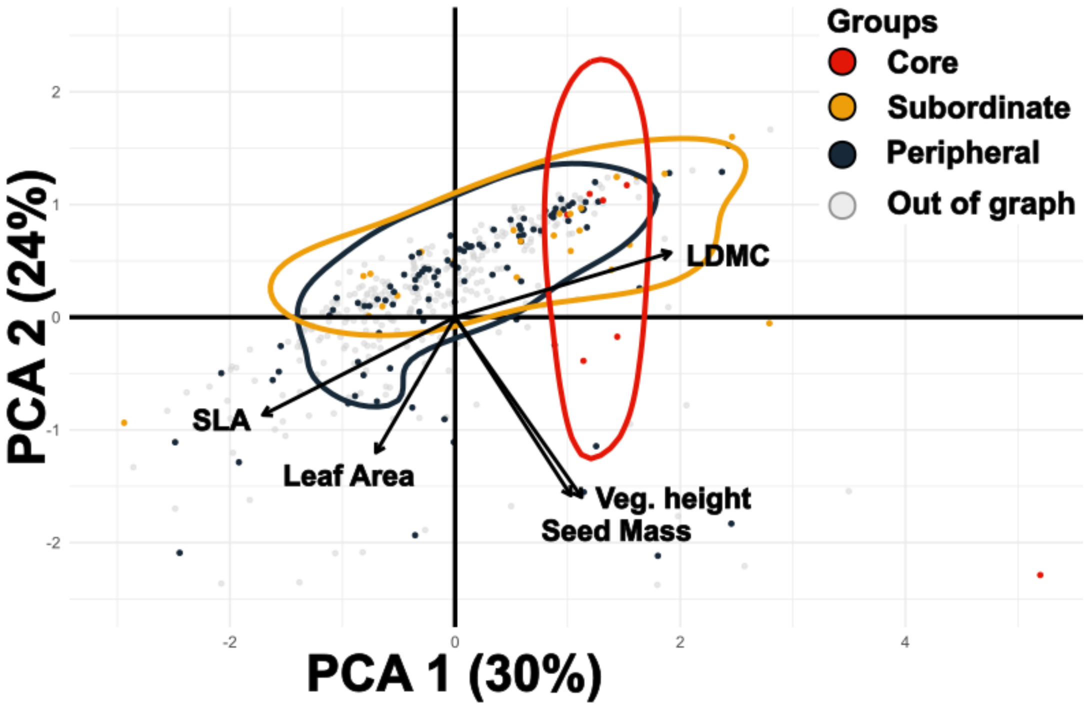


**Figure 9:** Principal Component Analysis (PCA) of the 422 plant species with measured trait data. Arrows indicate the direction and contribution of each trait (SLA, Leaf Area, LDMC, Seed Mass, and Vegetative Height) to the first two principal components. Contour lines represent the trait space occupancy density of the three groups identified in the regional spatial association network: core (red), subordinate (orange), and peripheral (dark blue). Species with no significant spatial associations are shown as grey points.

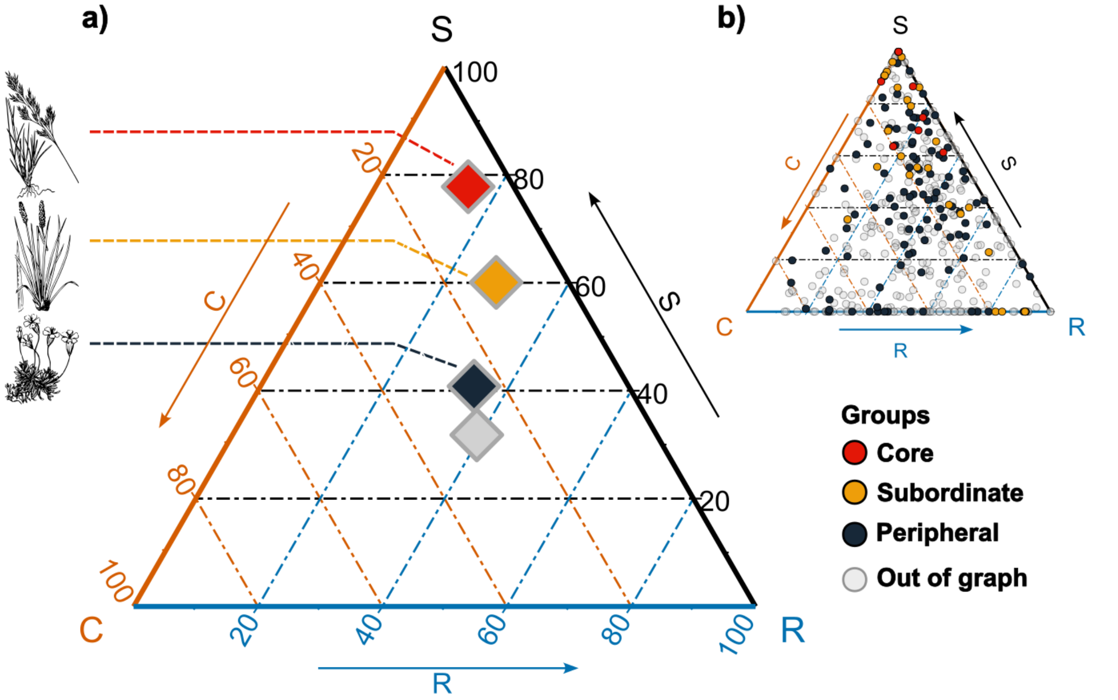


**Figure 10:** CSR strategies of species groups in the regional spatial association network. a) Centroids of relative proportions (%) of Competitive (C), Ruderal (R), and Stress-tolerant (S) strategies for the three network groups: core (red), subordinate (orange), and peripheral (dark blue). Species with no significant associations are shown in grey. Illustrations on the left depict representative species. b) CSR strategies of all 422 plant species with measured trait data. Each point represents one species, coloured according to its group in the regional spatial association network.

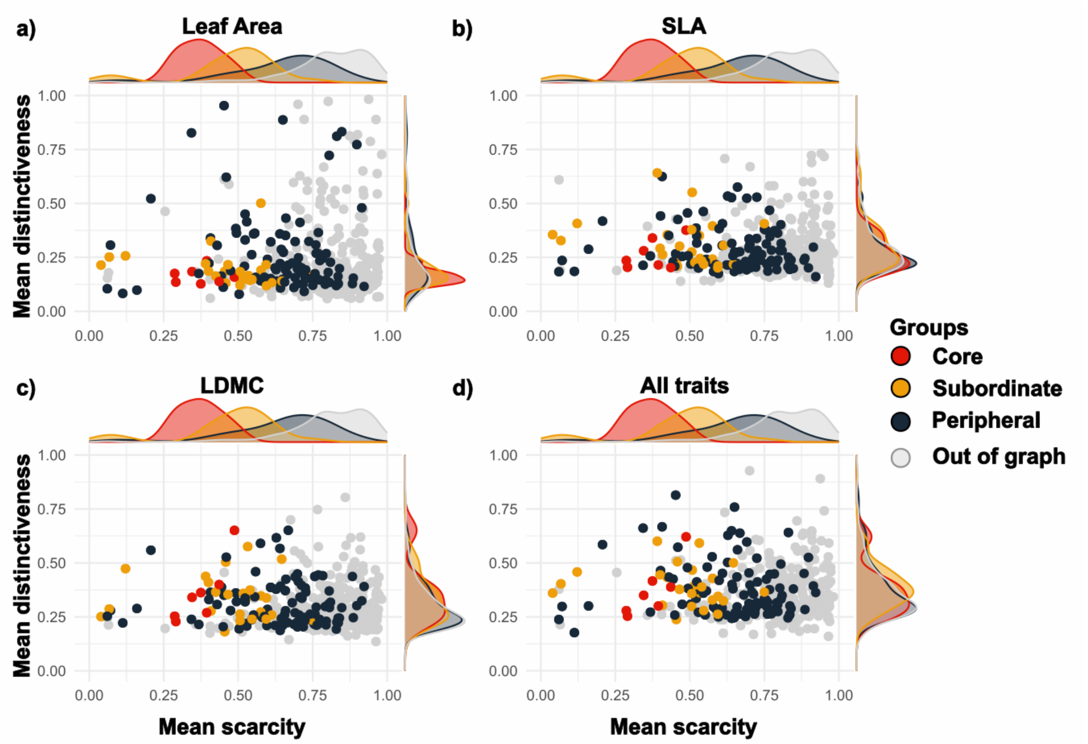


**Figure 11:** Biplots of species' mean functional distinctiveness against mean scarcity, with measured traits data only. Panels a) to c) display trait-specific distinctiveness for a) Leaf Area, b) SLA, and c) LDMC. Panel d) shows distinctiveness considering all traits combined. Species are coloured according to their group in the regional spatial association network: core (red), subordinate (orange), peripheral (dark blue), and non-associated species (grey). Marginal distributions are indicated on the sides.

### S4.2: Functional rarity

In the main text, for readability, we only showed the functional distinctiveness of Leaf Area, SLA, LDMC and the combination of traits. Here we show the results for the additional traits considered in this study (Seed Mass and Vegetative height, Figure 12).

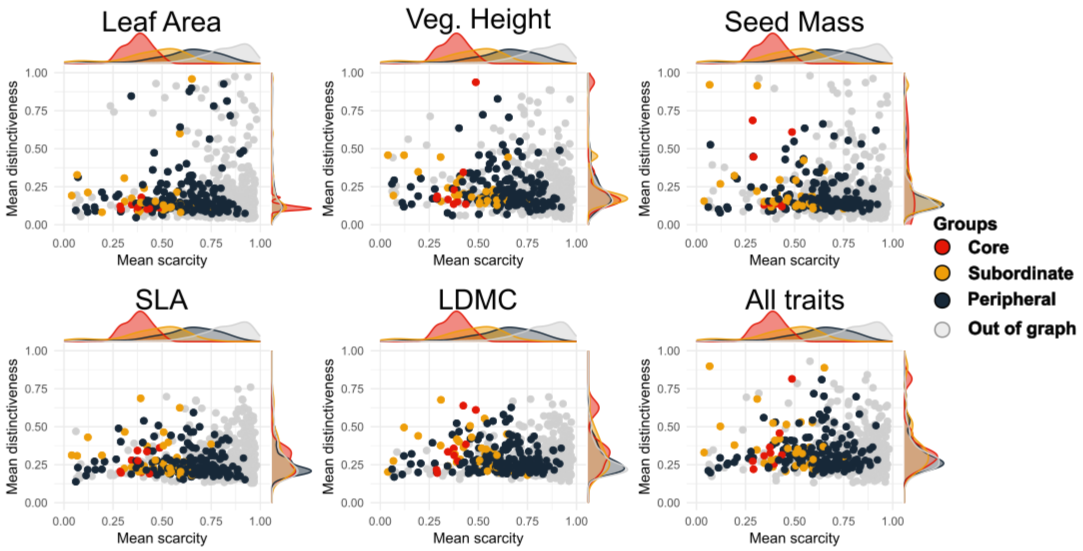


**Figure 12:** Biplots of species' mean functional distinctiveness against mean scarcity. Species are coloured according to their group in the regional spatial association network: core (red), subordinate (orange), peripheral (dark blue), and non-associated species (grey). Marginal distributions are indicated on the sides.

## S4.3: CSR strategies

We compared the distribution of the different groups along the stress axis with a Kruskal-Wallis test, followed by a Dunn test to investigate pairwise difference.

Difference in strategies were marginally significant between core and subordinates species ($p = 0.08$). In contrast, all the other pairwise comparison showed a significant difference (Figure 13).

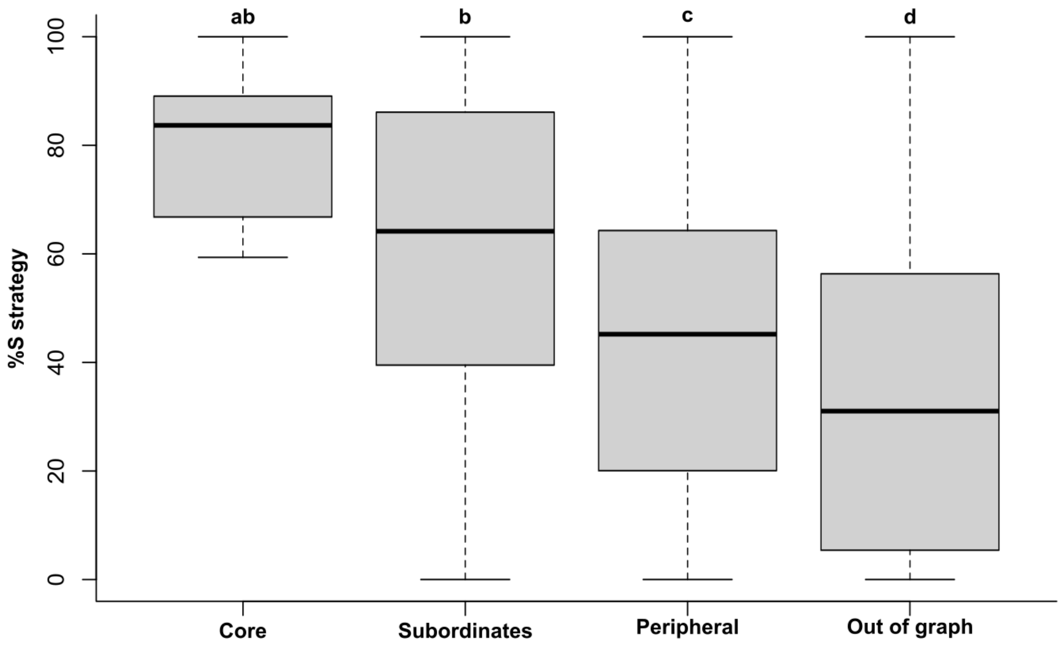


**Figure 13:** Differences in stress-tolerance (S) strategy among species groups. Letters above the boxes indicate statistically significant groupings based on pairwise comparisons (groups sharing a letter are not significantly different).

# Appendix S5: Environmental covariates

## S5.1: Soil data variation

Soil conditions were measured in situ by sampling three 2 × 2 m subplots within each plot. In each subplot, approximately 1 kg of soil was collected at a depth of  15 cm (excluding litter), and soil physicochemical properties were measured separately for each sample. For each plot, the three measurements were then averaged to obtain a single soil value. Because Alpine soils can exhibit substantial heterogeneity even at small spatial scales, we assessed within-plot variability by plotting the intra-plot variability against the calculated means(Figure 14)

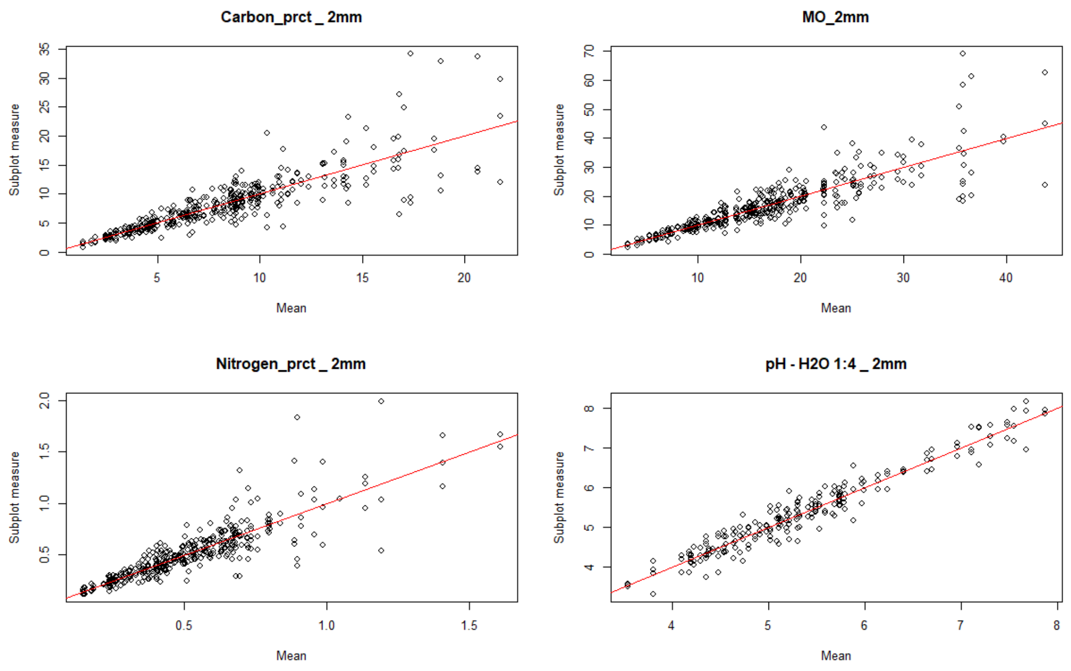


**Figure 14:** Plot-level mean soil values plotted against within-plot individual measure. The red line indicates the 1:1 isocline. A separate panel is shown for each soil variable used in the main text.

### S5.2: Species richness and local abundance Vs. climatic variable

We verify the potential for confounding variable, we tested whether there was any correlation between species richness, diversity, or the number of individual records and the environmental covariates (Figure 15).

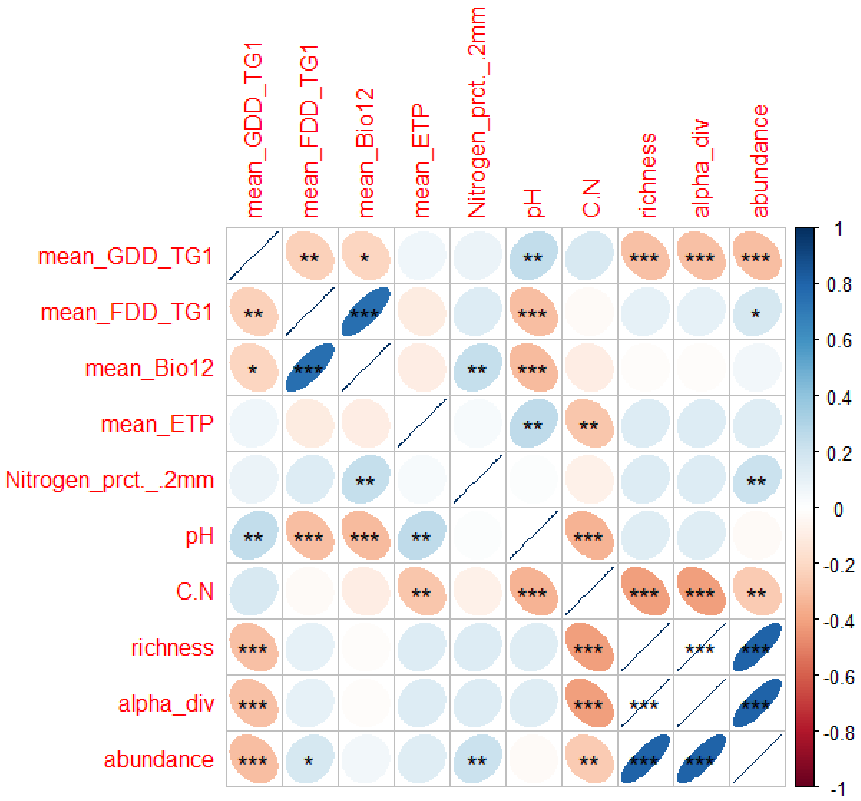


**Figure 15:** Correlation among environmental covariates and species richness, diversity and the number of individual records. Asterisks mark the different significativity levels ($***: p < 0.001$; $**: p < 0.01$; $*: p < 0.05$)

### S5.3: Alternative model, selecting precipitation over FDD

**Table 6:** Model predicting the number of spatial associations from local number of individuals alone vs. from local number of individuals combined with abiotic factors, including interactions (e.g., Nb of individuals:ETP). Signif. codes: *** $p < 0.001$, ** $p < 0.01$, * $p < 0.05$, n.s: not significant.

| Model | Coefficients | | | $R^2$ partition | Adjusted $R^2$ |
|---|---|---|---|---|---|
| *Nb of individuals* | Nb of individuals | $1 \cdot 10^{-3}$ | *** | 100% | 31% |
| | Nb of individuals | $2.7 \cdot 10^{-3}$ | n.s | 19.9% | |
| | pH | $-2.4 \cdot 10^{-1}$ | *** | 14.2% | |
| | N% | 1.6 | ** | 12.2% | |
| *Nb of individuals ∗ abiotic* | ETP | $5.4 \cdot 10^{-2}$ | n.s | 1.8% | 46% |
| | mean annual precipitation | $-5.4 \cdot 10^{-4}$ | n.s | 2.6% | |
| | Nb of individuals:ETP | $-7.1 \cdot 10^{-5}$ | n.s | 12.7% | |
| | Nb of individuals:precipitation | $6 \cdot 10^{-7}$ | n.s | 14.6% | |
| | Nb of individuals:N% | $-1.2 \cdot 10^{-3}$ | n.s | 16.6% | |
| | GDD | $-1.7 \cdot 10^{-4}$ | n.s | 5.8% | |

# Appendix S6: Consortium ORCHAMP

## S6.1: Institutions involved in ORCHAMP

ORCHAMP is a consortium gathering a large range of actors: national and regional park managers, botanical conservatory experts, natural area conservatory managers, association, researchers from universities and research institutions. The project is led by the LECA (Laboratoire d'Écologie Alpine), located in Grenoble.

For additional information please visit our website: https://orchamp.osug.fr/home or contact us: orchamp@univ-grenoble-alpes.fr

- **LECA** - Laboratoire d'Écologie Alpine; Univ. Grenoble Alpes, Univ. Savoie Mont Blanc, CNRS, LECA, F-38000 Grenoble, France
  `https://leca.osug.fr/`

- **EDYTEM** - Environnements, DYnamiques et TErritoires de la Montagne; Univ. Savoie Mont Blanc, Univ. Grenoble Alpes, CNRS, EDYTEM, Chambéry, France
  `http://edytem.univ-savoie.fr/`

- **INRAE LESSEM** - Laboratoire Ecosystèmes et Sociétés En Montagne; Univ. Grenoble Alpes, LESSEM, INRAE, Grenoble, France
  `https://www6.lyon-grenoble.inrae.fr/lessem/`

- **IMBE** - Institut Méditerranéen de Biodiversité et d'Écologie marine et continentale; Aix Marseille Univ, Avignon Univ, CNRS, IRD, Marseille, France
  `https://www.imbe.fr/l-unite.html`

- **Jardin du Lautaret** - Univ. Grenoble Alpes, CNRS, Jardin du Lautaret, F-38000 Grenoble, France https://www.jardinalpindulautaret.com

- **CEFE** - Centre d'Ecologie Fonctionnelle et Evolutive; Univ. Montpellier, CNRS, EPHE, IRD, Montpellier, France
  `https://www.cefe.cnrs.fr/fr/`

- **CEN** - Centre d'Études de la Neige; Univ. Grenoble Alpes, Université de Toulouse, Météo-France, CNRS, Centre National de Recherches Météorologiques, Grenoble, France
  `https://www.umr-cnrm.fr/spip.php?rubrique85`

- **INRAE ECODIV** - Laboratoire Etude et Compréhension de la bioDIVersité; Univ. Rouen - Normandie, INRAE, ECODIV, Rouen, France
  `http://ecodiv.univ-rouen.fr/fr`

- **Laboratoire de Géologie de l'ENS**; CNRS, ENS, Univ. PSL, Paris, France
  `http://www.geologie.ens.fr/spiplabocnrs/`

- **INRAE URFM** - UR Ecologie des Forêts Méditerranéennes; INRAE, Avignon, France
  https://ecologie-des-forets-mediterraneennes.paca.hub.inrae.fr/
- **INRAE UEFM** - UE Entomologie et Forêt Méditerranéenne; INRAE, Avignon, France
  https://uefm.paca.hub.inrae.fr/
- **DYNAFOR** - Dynamiques et Écologie des Paysages Agroforestiers; INRAE, INP, Castanet Tolosan, France
  https://www.dynafor.fr/
- **SETE** - Station d'Ecologie Théorique et Expérimentale; Univ. Paul Sabatier, CNRS, Moulis, France
  https://sete-moulis-cnrs.fr/fr/
- **CRBE** - Centre de Recherche sur la Biodiversité et d'Environnement; Université de Toulouse, IRD, INP, CNRS, Université Toulouse 3 Paul Sabatier (UT3); Toulouse, France
  https://crbe.cnrs.fr/
- **GEODE** - Géographie de l'Environnement; Maison de la recherche Univ. Jean Jaurès, CNRS, Toulouse, France
  https://geode.univ-tlse2.fr/
- **PatriNat** - Centre d'expertise et de données sur le patrimoine naturel; OFB, MNHN, CNRS, IRD, Paris, France
  https://www.patrinat.fr/fr
- **AR+I** - Andorra Recerca + Innovació, Sant Julià de Lòria, Andorre
  https://www.ari.ad/en
- **LEM** - Laboratoire d'Ecologie Microbienne; Univ. Lyon 1, CNRS, INRAe, VetAgro Sup, Lyon, France
  https://www.ecologiemicrobiennelyon.fr/
- **Eco&Sols** - INRAE, IRD, CIRAD, Institut Agro, Montpellier, France
  https://www.umr-ecosols.fr/
- **ZA Alpes** - Zone Atelier Alpes, Grenoble, France
  https://za-alpes.osug.fr/
- **ZA PYGAR** - Zone Atelier Pyrénées GARonne, Toulouse, France
  https://pygar.omp.eu/
- **CBN Alpin** - Conservatoire Botanique National Alpin, Domaine de Charance, 05000 Gap, France
  http://www.cbn-alpin.fr/
- **CBN Med** - Conservatoire Botanique National Méditerranéen, Hyères, France
  http://www.cbnmed.fr/

- **CBN PMP** - Conservatoire Botanique National Pyrénées et Midi-Pyrénées, Bagnère-de-Bigorre, France

  http://cbnpmp.blogspot.com/

- **PN des Ecrins** - Parc National des Ecrins & Réserve intégrale du Lauvitel, Gap, France

  http://www.ecrins-parcnational.fr/

- **PN du Mercantour** - Parc National du Mercantour, Nice, France

  http://www.mercantour-parcnational.fr/fr

- **PN de la Vanoise** - Parc National de la Vanoise, Chambéry, France

  http://www.vanoise-parcnational.fr/fr

- **PN des Pyrénées** - Parc National des Pyrénées, Tarbes, France

  https://www.pyrenees-parcnational.fr/fr

- **PNR du massif des Bauges** - Parc naturel régional du massif des Bauges & Géoparc mondial UNESCO, Le Châtelard, France

  http://www.parcdesbauges.com/fr/

- **PNR de Chartreuse** - Parc naturel régional de Chartreuse, 11 Place de la mairie, 38380 Saint-Pierre-de-Chartreuse, France

  http://www.parc-chartreuse.net/

- **PNR du Queyras** - Parc naturel régional du Queyras & Réserve Naturelle Nationale de Ristolas - Mont Viso, 3580 route de l'Izoard, 05350 Arvieux, France

  https://www.pnr-queyras.fr/

- **PNR du Mont-Ventoux** - Parc naturel régional du Mont-Ventoux, Carpentras, France

  https://www.parcduventoux.fr/

- **RNR du Massif de Saint-Barthélemy** - Réserve Naturelle Régionale du Massif de Saint-Barthélemy, Montségur, France

  https://ariegenature.fr/rnr/

- **RNR d'Aulon – CEN Occitanie, La Frênette et Commune d'Aulon**

  https://www.cen-occitanie.org

- **Réserves Naturelles Catalanes - RNN de la vallée d'Eyne** - Fédération des Réserves naturelles catalanes, 9 rue de Mahou 66500 Prades

  https://www.reserves-naturelles-catalanes.org/

- **OFB** - Office français de la biodiversité, gestionnaire de la Réserve nationale de chasse et de faune sauvage d'Orlu
  https://www.ofb.gouv.fr/les-reserves/la-reserve-nationale-de-chasse-et-de-faune-sauvage-dorlu

- **Observatoire de la montagne, Commune d'Orlu**, Orlu, France
  `https://www.vallee-orlu.com/fr/la-reserve-nationale.html`

- **Adyu l'Ome**, Orlu, France
  `http://www.adyulome.com/`

- **ANA-CEN Ariège** - Conservatoire d'Espace Naturel Ariège, Alzen, France
  `https://ariegenature.fr/`

- **Géoparc du Chablais** - Géoparc mondial UNESCO du Chablais, Thonon-les-Bains, France
  `https://www.geoparc-chablais.com/`

- **Asters-CEN74** - Conservatoire d'espaces naturels de Haute-Savoie & Réserve Naturelle de Sixt - Fer à Cheval/Passy, Sixt-Fer-à-Cheval, France
  `http://www.cen-haute-savoie.org/`

- **CREA Mont-Blanc** - Centre de Recherches sur les Écosystèmes d'Altitude, Chamonix, France
  `https://creamontblanc.org/fr`

- **Natura 2000 Clarée** - Névache, France
  `https://inpn.mnhn.fr/site/natura2000/FR9301499`
  `https://www.nevache.fr/natura-2000/`

- **Natura 2000 Dévoluy-Durbon-Charance-Champsaur SMIGIBA** - Syndicat Mixte de Gestion Intercommunautaire du Buëch et de ses Affluents Veynes, France
  `http://hautes-alpes.n2000.fr/devoluy`
  `http://www.smigiba.fr/`

- **Grenoble-Alpes Métropole** - Grenoble, France
  `https://www.grenoblealpesmetropole.fr/`

- **ONF** - Office National des Forêts - Grenoble
  ag.isere@onf.fr

## S6.2: ORCHAMP Consortium (contact persons are in italics)

- **LECA**: *Wilfried Thuiller, Amélie Saillard*, Louise Boulangeat (2018–2021), Manon Bounous (2018), Irene Calderon-Sanou, Philippe Choler, Camille Desjonquères, Arnaud Foulquier, Ludovic Gielly, Priscilla Godfroy (2017–2019), Romain Goury, Maya Guéguen, Nicolas Le Guillarme, Clément Lionnet (2018–2023), Chloé Mahieu, Camille Martinez-Almoyna, Marc Ohlmann (2016-deceased in 2023),

Gabin Piton (2016–2019), Julien Renaud, Matthias Rohr, Guillaume Terpereau (Student 2021), Tristan Ubaldi (Student 2019)

- **EDYTEM**: *Jérome Poulenard*, Nicolas Bonfanti, Norine Khedim (2018–2022), Emmanuel Malet, Lise Marchal (2019–2023), Erwan Messager, Yves Perrette
- **INRAE LESSEM**: *Georges Kunstler*, *Vincent Breton*, Laurent Berges, Nathan Daumergue, Adeline François, Sophie Labonne (retired in 2023), Laureline Leclerc (Student 2022), Eric Mermin, Jean-Matthieu Monnet (2018–2019), Yoan Paillet, Mathias Pires, Pascal Tardif (2016-retired in 2023)
- **IMBE**: *Frédéric Guiter, Lenka Brousset*, Cécile Albert, Armin Bischoff, Manuel Cartereau, Cécile Chemin, Emmanuel Corcket, Amandine Gasc, Raphaël Gros, Frédéric Guibal, Frédéric Médail (2018–2019), Eric Meineri, Jean-Philippe Mévy, Alexandre Millon (2018–2020), Pascal Mirleau, Daniel Pavon, Yoann Pinguet, Hervé Ramone, Caroline Rocher, Arne Saatkamp, Brigitte Talon
- **Jardin du Lautaret**: *Jean-Gabriel Valay*, *Jérôme Forêt (since 2023)*, Rolland Douzet, Lucie Liger, Maxime Rome, Pascal Salze
- **CEFE**: Jean-François David, Cyrille Violle
- **CEN**: *Samuel Morin*, Matthieu Vernay, Matthieu Lafaysse
- **ECODIV**: *Lauric Cécillon* (2016–2022)
- **Laboratoire de Géologie de l'ENS**: *Lauric Cécillon* (2016–2022), Laure Soucémarianadin (till 2022)
- **INRAE URFM**: *Bruno Fady*, William Brunetto, Florence Courdier, Frédéric Jean, Nicolas Mariotte
- **INRAE UEFM**: *Jean Thévenet*, Marianne Corréard
- **DYNAFOR**: Laurent Larrieu, *Antoine Brin*, Laurent Raison, Célia Sirami, Catherine Bonnet, Jérôme Willm, Alexis Carteron
- **SETE**: Maxime Cauchoix
- **CRBE**: *Jérôme Murienne*, Gabrielle Martin, Renan Destrade, Uxue Suescun
- **GEODE**: Marie-Claude Bal, Mélanie Saulnier
- **PatriNat**: *Olivier Delzons*, Philippe Gourdain, Aurélie Lacoeuilhe
- **AR+I**: Benjamin Komac
- **LBBE**: Vincent Miele
- **LEM**: Juliana Almario, Lauren Gillespie

- **Eco&Sols**: *Mickaël Hedde*, Matthias Brand, Thomas Gelis, Nicolas Hénon, Cyril Versavel, Luna Vion-Guibert
- **ZA Alpes**: *Mathilde Ratouis*, Isabelle Arpin (till 2024), Renaud Jaunatre (since 2024), Marc Langenbach (since 2024), Erwan Messager (since 2024), Tamara Münkemüller (since 2024), Jérôme Poulenard (till 2024)
- **ZA PYGAR**: Arnaud Elger
- **CBN Alpin**: *Bertrand Liénard, Sylvain Abdulhak, Léa Bizard*, Gilbert Billard, Pauline Debay, Luc Garraud, Thomas Legland, Baptiste Merhan, Mathieu Michoulier, Gilles Pache, David Paulin, Thomas Sanz, Jérémie Van Es
- **CBN Med**: *Virgile Noble*, Pauline Bravet, Benoît Offerhaus, Henri Michaud, Maëlle Le Berre, Mathias Pires (till 2023), Julien Ugo, Marion Girardier
- **CBN Pyrénées et Midi-Pyrénées**: Jocelyne Cambecèdes, *Ludovic Olicard*, Michaël Douette, Anne Paris, Gilles Corriol
- **PN des Ecrins & RI du Lauvitel**: *Richard Bonet*, François Couilloud, Cédric Dentant, Damien Combrisson, Yoann Bunz, Jérôme Forêt (based in Jardin du Lautaret since 2023)
- **PN du Mercantour**: *Clémentine Assmann* (since 2024), Sébastien Honoré, Mathieu Krammer, Benoit Labigand, Marie-France Leccia (till 2024), Jérôme Mansons, Nathalie Siefert (till end 2023)
- **PN de la Vanoise**: *Vincent Augé*, *Joël Blanchemain*, Anne Bello (since 2024), Thierry Delahaye, Nicolas Gomez (since 2024), Franck Parchoux (2017–2021)
- **PN des Pyrénées**: *Pierre Lapenu*, *Olivier Jupille*, Jérémy Bauwin, Nils Paulet, Océane Pasquet, Sylvain Rollet,
- **PNR du massif des Bauges**: *Jean-François Lopez*, Richard Cousin
- **PNR de Chartreuse**: *Bastien Moisan, Laure Belmont*, Jessica Bruggeman (2019–2022)
- **PNR du Queyras & RN Ristolas-Mont-Viso**: *Anne Goussot, Pierpaolo Brena*, Alain Bloc (retired in 2023), Nicolas Tenoux
- **PNR du Mont-Ventoux**: Baptiste Montesinos, Anthony Roux
- **RNR du Massif de Saint-Barthélemy**: *Laurent Servière*
- **RNR d'Aulon**: *Cyril Marmoex*, Loyann Boy, Maëlle Benureau, Coline Carré, Lucyna Loriot (student 2024)
- **Réserves Naturelles Catalanes - RNN de la vallée d'Eyne**: Josep Parera Casas, Céline Quelennec

- **OFB - gestionnaire de la RNCFS d'Orlu**: *Xavier Rozec*
- **Observatoire de la montagne, Commune d'Orlu**: *Christophe Lhez*
- **Adyu l'Ome**: Pierre Guiton, Thérèse Sabadie
- **ANA-CEN Ariège**: *Laurent Servière*
- **Géoparc du Chablais**: *Sophie Justice*
- **ASTERS & RN Sixt-Passy**: *Carole Birck*, Olivier Billant, Jean-José Richard-Pomet
- **CREA**: *Anne Delestrade*, Bradley Carlson (2017–2023), Colin Van Reeth, Jeremy Froidevaux (since 2024)
- **N2000 Clarée**: *Laure Vuinée*
- **Natura 2000 Dévoluy-Durbon-Charance-Champsaur & SMIGIBA**: *Eric Hustache*
- **Grenoble-Alpes Métropole**: *Alexandre Mignotte*, Pierre-Eymard Biron (retired in 2021), Yann Kohler
- **ONF**: Carole Desplanque, Laurent Lathuillière
- **Indépendants**: Jean-Marie Dupont (Apexe), Françoise Laigneau, Christophe Perrier (till 2020), Olivier Senn, Alexandre Pailhé-Belair (Berger, estive d'Aulon), Alexis James (Berger, estive d'Aulon)

### S4.3: ORCHAMP funding

Each institution involved in the consortium is co-funding the project either through in-kind funding or participation to specific projects.

**ANR - Agence Nationale de la Recherche**: GlobNets (ANR-16-CE02-0009), Origin-Alps (ANR-16-CE93-004), TransAlps (ANR-16-CE02-0009) & EcoNet (ANR-18-CE02-0010-01)

**ANR "Investissement d'Avenir"**: Trajectories (ANR-15-IDEX-02), Montane (OSUG@2020: ANR-10-LAB-56), Idex UGA (ANR-15-IDEX-02); MIAI@Grenoble Alpes (ANR-19-P3IA-0003)

**AFB - Agence Française pour la Biodiversité**: Sentinelles des Alpes 2018–2019

**OFB - Office français de la biodiversité**: Sentinelles des Alpes 2020–2022, Sentinelles des Alpes 2023, Sentinelles des Alpes 2024

**AURA - Région Auvergne-Rhône-Alpes**: CBNA regional convention

**SUD-PACA - Région Sud Provence-Alpes-Côte d'Azur**: Support to CBNA and CBNMED

**LTSER ZAA - Zone Atelier Alpes** (CNRS, INRAE; membre de eLTER)

**ISÈRE - Le Département**: Appel à projets Biodiversité 2020

**OSUG - Service d'observation**: Appel à projets 2020

**ECCOREV - Ecosystèmes Continentaux et Risques Environnementaux**: Appel à projets 2019

**Interreg Alcotra FEDER** - PITEM Biodiv'ALP 2019-2023 PS3. - GEBIODIV

**Interreg POCTEFA**: FLORAPYR3D 2024–2026

**BIOSEFAIR INRAE**: SICCCUB 2021–2023

**Région Occitanie**: PAACTe Région Occitanie, 2022–2024

**Interreg Alcotra FEDER**: PITEM Biodiv'ALP

**CNRS initiative EC2CO** - Projet Microphos AAP 2021–2023

**Other local fundings**: Parc National des Ecrins (PNE), Réserve intégrale du Lauvitel, Parc National du Mercantour (PNM), Grenoble Alpes Métropole, Agence de l'eau Rhône-Méditerranée Corse (AERMC), Electricité de France (EDF), Mairie du Dévoluy, Institut de Radioastronomie Millimétrique (IRAM), Communauté de communes de la vallée de Chamonix Mont-Blanc, Fonds européen agricole pour le développement rural (FEADER).

## References

Benjamini, Y. and Hochberg, Y. (1995). Controlling the false discovery rate: a practical and powerful approach to multiple testing. *Journal of the Royal statistical society: series B (Methodological)*, 57(1):289–300.

Chiquet, J., Donnet, S., and Barbillon, P. (2024). *sbm: Stochastic Blockmodels.* R package version 0.4.7.

Csárdi, G., Nepusz, T., Traag, V., Horvát, S., Zanini, F., Noom, D., and Müller, K. (2025). *igraph: Network Analysis and Visualization in R.* R package version 2.1.3.

Delmas, E., Besson, M., Brice, M.-H., Burkle, L. A., Dalla Riva, G. V., Fortin, M.-J., Gravel, D., Guimarães, P. R., Hembry, D. H., Newman, E. A., Olesen, J. M., Pires, M. M., Yeakel, J. D., and Poisot, T. (2019). Analysing ecological networks of species interactions: Analyzing ecological networks. *Biological Reviews*, 94(1):16–36.

Erdos, P., Rényi, A., et al. (1960). On the evolution of random graphs. *Publ. math. inst. hung. acad. sci*, 5(1):17–60.

Snijders, T. A. and Nowicki, K. (1997). Estimation and prediction for stochastic blockmodels for graphs with latent block structure. *Journal of classification*, 14(1):75–100.

Watts, D. J. and Strogatz, S. H. (1998). Collective dynamics of 'small-world'networks. *nature*, 393(6684):440–442.
